# Association of Dietary Choline Intake With Incidence of Frailty: A Nationwide Prospective Cohort Study From China

**DOI:** 10.1002/jcsm.13796

**Published:** 2025-04-06

**Authors:** Lian‐hong Chen, Jian‐feng Zhong, Ying‐ying Niu, Cheng‐ping Li, Jing Li, Zhi‐quan Diao, Hao‐yu Yan, Miao Xu, Wen‐qi Huang, Zhi‐tong Xu, Chang Su, Dan Liu

**Affiliations:** ^1^ Department of Public Health and Preventive Medicine, School of Medicine Jinan University Guangzhou Guangdong China; ^2^ National Institute for Nutrition and Health Chinese Center for Disease Control and Prevention Beijing China; ^3^ Key Laboratory of Trace Element Nutrition National Health Commission Beijing China; ^4^ Department of Epidemiology, School of Public Health Southern Medical University Guangzhou Guangdong China

**Keywords:** cohort study, dietary choline, frailty

## Abstract

**Background:**

Emerging evidence suggests that dietary choline is a modifiable nutritional factor linked to various health outcomes. However, most existing studies have focused on isolated health conditions, lacking a comprehensive assessment of overall health status. This study aimed to investigate the association between total dietary choline intake and frailty incidence among Chinese adults, considering its derivatives, soluble forms (water‐soluble and lipid‐soluble) and food sources (animal‐derived and plant‐derived).

**Methods:**

Participants without frailty at baseline were enrolled from the China Health and Nutrition Survey (CHNS), with follow‐up from 2004 to 2016. Dietary intake was assessed using three consecutive 24‐h dietary recalls to estimate total dietary choline intake, its derivatives, soluble forms and food sources. Frailty status was evaluated using a frailty index (FI), with frailty defined as an FI > 0.21. Cox proportional hazards regression and restricted cubic splines were used to analyse the associations between dietary choline intake and frailty incidence.

**Results:**

A total of 10 310 participants (mean age: 46.4 years [SD: 14.5]; 52.6% female) were eligible. During a median follow‐up of 6.1 years, 1150 incident frailty cases were recorded. Cox models with penalized splines showed an L‐shaped association between total dietary choline intake and frailty incidence. Compared with participants in the lowest quartile of total choline intake, those in the 2nd to 4th quartiles had lower odds of frailty, with hazard ratios (HRs) of 0.84 (95% CI: 0.71, 0.98), 0.80 (95% CI: 0.67, 0.95) and 0.75 (95% CI: 0.61, 0.93), respectively. Intake of lipid‐soluble choline in the 2nd to 4th quartiles was associated with an 18% (HR: 0.82; 95% CI: 0.69, 0.98) to 23% (HR: 0.77; 95% CI: 0.63, 0.95) reduction in the odds of frailty. Participants in the 3rd to 4th quartiles of phosphatidylcholine intake exhibited 19% (HR: 0.81; 95% CI: 0.68, 0.96) to 23% (HR: 0.77; 95% CI: 0.63, 0.94) lower odds of frailty. Choline intake from plant‐derived food sources was significantly associated with reduced odds of frailty (HR: 0.83; 95% CI: 0.69, 0.99).

**Conclusions:**

Moderate to high dietary choline intake (171.00–464.99 mg/day), particularly phosphatidylcholine (145.20–304.93 mg/day), may be associated with reduced odds of frailty.

## 1 Introduction

Frailty, an age‐associated clinical condition, is characterized by diminished physiological capacities, compromised biological mechanisms and heightened vulnerability to adverse health outcomes, such as morbidity, mortality, cognitive impairment, physical disability and increased healthcare expenditures [1]. A meta‐analysis revealed a frailty prevalence of 12%, reaching 24% based on the deficit accumulation model; for pre‐frailty, these figures were 46% and 49%, respectively [2]. Frailty has emerged as a significant public health concern amid global population aging [3]. However, frailty is a dynamic transition that is demonstrated to possess mutability and reversibility [4]. Therefore, identifying modifiable risk factors and developing interventions to prevent, delay or reverse frailty progression is crucial for public health [5].

Several studies suggest that nutrient substances are potentially modifiable contributors to the risk and prevention of frailty [6]. Choline, an essential nutrient, is crucial for the biosynthesis of neurotransmitters and the formation of phospholipids integral to cell membrane structure [7]. Previous studies have demonstrated beneficial associations between choline and multiple health outcomes. The Framingham Heart Study showed that low choline intake is associated with an elevated risk of dementia and Alzheimer's disease [8]. Another cohort study suggested that choline and choline‐rich foods have protective effects against hypertension [9]. However, these studies typically focus on singular health outcomes, limiting the evaluation of overall health status. Most studies also focused exclusively on choline, overlooking its subtypes. Moreover, no cohort studies have examined the association between choline and frailty. Most epidemiological research on frailty excludes individuals under the age of 65, but frailty may be significant in the adult population as well [10].

Therefore, the present study aimed to explore the potential associations of choline and its subcategories intake and source (lipid‐ and water‐soluble, animal‐ and plant‐derived) with the incidence of pre‐frailty and frailty in Chinese adults. Our hypothesis posited that the choline consumption may have varying effects on the occurrence of pre‐frailty and frailty within this demographic.

## 2 Methods

### 2.1 Study Design and Participants

The China Health and Nutrition Survey (CHNS) was a nationwide household‐based prospective cohort study, and the details of the study protocol have been described elsewhere [S1]. Briefly, the CHNS was established in 1989 and included participants of diverse ages from nine provinces and three directly controlled municipalities (added in 2011). By 2011, the regions represented in the CHNS comprised 47% of China's overall population. The cohort employed a multistage random cluster sampling design to ensure representativeness. The survey focused on examining social demographic factors, nutritional and dietary status, physical activity and health outcomes.

This study used data from the 2004, 2006, 2009 and 2011 waves of the CHNS, as the survey has provided more reliable dietary data since 2004. The following participants were excluded: (1) 3807 participants who were less than 18 years old at the baseline; (2) 188 participants who were pregnant; (3) 2037 participants who were without complete dietary data at the baseline; (4) 2422 participants with missing demographics, lifestyle or community index data; (5) 89 participants with implausible energy intake information (male: < 800 kcal/day or > 6000 kcal/day; female: < 600 kcal/day or > 4000 kcal/day); and (6) 465 participants with missing more than 20% of frailty index (FI) items (i.e., six or more items) at baseline [S2]. A total of 13 676 participants had a valid FI at baseline. The study was divided into three parts. First, we analysed the association between dietary choline intake and incident frailty. Participants with frailty (FI > 0.21) at baseline (*n* = 1032) or those without follow‐up data (*n* = 2334) were excluded, resulting in a final sample of 10 310 participants. Second, we investigated the association between dietary choline intake and incident pre‐frailty. Participants with (pre‐) frailty (FI > 0.1) at baseline (*n* = 4746), those who without follow‐up data (*n* = 1708) or those who developed new‐onset frailty during follow‐up (*n* = 253) were excluded, leaving a total of 6969 participants. Third, we analysed the association between dietary choline intake and incident (pre‐) frailty. Participants with (pre‐) frailty (FI > 0.1) at baseline (*n* = 4746) or those without follow‐up data (*n* = 1708) were excluded, resulting in 7222 participants included (Figure S1).

The CHNS is a collaborative project between the Carolina Population Center (CPC), University of North Carolina at Chapel Hill and the China National Institute of Nutrition and Food Safety at the Chinese Center for Disease Control and Prevention. Written informed consent was obtained from each CHNS participant, and the study was approved by the institutional review boards at the University of North Carolina at Chapel Hill (NC, USA) and the China National Institute of Nutrition and Food Safety at the Chinese Center for Disease Control and Prevention (Beijing, China).

### 2.2 Assessment of Dietary Choline

The dietary data were obtained through three consecutive 24‐h dietary recalls (24h‐DRs) at the individual level, coupled with a household‐level weighting method of food inventory across the same three‐day period for each survey round [11]. The three consecutive days comprised two weekdays and one weekend day. The accuracy of 24h‐DRs in assessing energy and nutrient intake had been validated [12]. Well‐trained interviewers utilized food pictures and models to meticulously record the type and quantity of foods consumed daily. Participants self‐reported their consumption of all foods over the preceding three days. Due to the lack of available data on the choline and betaine content in the Chinese Food Composition Tables (CFCT), the values for these nutrients in the diet are based on the USDA database [13, 14]. Total choline is composed of phosphatidylcholine, sphingomyelin, free choline, glycerophosphocholine and phosphocholine. Lipid‐soluble choline included phosphatidylcholine and sphingomyelin, while water‐soluble choline included free choline, glycerophosphocholine and phosphocholine [15]. Animal‐ and plant‐derived choline intakes were calculated based on food sources. Dietary choline and betaine intakes were estimated by multiplying the quantity of each food item consumed by the nutrient content for a standard 100 g portion size. The average daily intake over a three‐day period was calculated for each participant based on their total intake.

### 2.3 Assessment of Frailty

The primary outcome of interest in this study was incident frailty. Frailty is commonly assessed using the FI, which is primarily based on the principle of cumulative deficits [16, 17]. Different studies, including CKB, CHARLS, CLHLS, the UK Biobank and NHANES, have developed FI that include varying items [18, 19, 20, 21, 22]. In this study, we developed an FI that includes 27 items across six dimensions: history of chronic diseases (based on self‐reported physician diagnoses), symptoms experienced in the last 4 weeks (based on self‐reported physician diagnoses), anthropometry indexes (measured by trained technicians using standard methods), health status (self‐reported), physical activity level (self‐reported) and sleep duration (self‐reported) (Table S1). More details on the items of FI are provided in Supporting Information: Supplement Methods 1. The FI is calculated as the ratio of the score of deficits exhibited by participants to the total score of potential deficits. For participants with missing data on certain deficits, we calculated the FI by excluding the absent items from both the numerator and the denominator. The FI score values range from 0 to 1, with a higher score indicating increased levels of frailty. According to the FI, participants were classified into three groups: non‐frailty (FI ≤ 0.1), pre‐frailty (0.1 < FI ≤ 0.21) and frailty (FI > 0.21) [23, 24]. In our study, the term (pre‐) frailty refers to the combined states of pre‐frailty and frailty [S3].

### 2.4 Assessment of Covariates

Demographics, lifestyle and anthropometric characteristic data were collected through structured questionnaires at baseline, including age, sex, nationality, residence, geographical region, marital status, education, household per capita annual income, medical insurance, drinking, smoking, drinking water source, cooking fuel type, sedentary behaviour, dietary intake of energy, protein, fat and carbohydrate, body mass index (BMI), hypertension, health infrastructure score, sanitation score and social services score. Drinking was assessed through the question, ‘Over the past year, what was your frequency and quantity of consumption of beer, wine, and liquor?’. The alcohol concentration was consistent with previous study: beer = 4%, wine = 10% and liquor = 38% (1 bottle = 600 mL, 1 Liang = 50 mL) [S4]. Following WHO guidelines [S5], low‐risk alcohol consumption was defined as < 21 g/day for females and < 41 g/day for males. We classified drinking into three categories: never (0 g/day); moderate (1–20 g/day for females, 1–40 g/day for males); and excess (≥ 21 g/day for females, ≥ 41 g/day for males). Smoking was categorized as never, previous and current. ‘Never’ was defined as a negative response to the question, ‘Have you ever smoked a cigarette?’. ‘Current’ was defined as an affirmative response to the question, ‘Do you still smoke?’. ‘Previous’ referred to participants who had smoked in the past but were not currently smoking. Based on a prior study, we classified electricity, liquefied petroleum gas and natural gas as clean fuel, while coal, charcoal, kerosene, wood, straw and sticks were classified as polluting fuel [S6]. Dietary intake (energy, protein, fat and carbohydrate) was estimated by calculating the product of the amount of each food item consumed and the nutrient composition for a standard portion size of 100 g, as referenced in the CFCT [S7‐S8]. BMI was calculated by dividing weight (kg) by height squared (m^2^). Hypertension was defined according to the following criteria: (1) mean systolic blood pressure (SBP) ≥ 140 mmHg and/or mean diastolic blood pressure (DBP) ≥ 90 mmHg; (2) self‐reported diagnosis of hypertension; (3) use of antihypertensive medication. Blood pressure was assessed three times with a standard mercury sphygmomanometer. We used the mean of measurements of SBP and DBP in our study. Scores for health infrastructure, sanitation and social services were used to assess urban features in China, based on an established urbanization scale [S9]. These scores were derived from established algorithms, with a maximum of 10 points allocated. More details on the covariates are provided in Supporting Information: Supplement Methods 2. Weight and height were measured by trained technicians utilizing standardized methods, while other covariates were self‐reported by participants.

### 2.5 Statistical Analysis

Baseline characteristics are presented as numbers (percentage %) for categorical variables and the mean (standard deviation [SD]) for continuous variables. We used chi‐square tests (categorical variables) and analysis of variance and nonparametric Kruskal–Wallis tests (continuous variables) to compare the baseline characteristics of participants according to quartiles of total choline intake.

Cox proportional hazards regression was utilized to estimate the hazard ratios (HRs) and corresponding 95% confidence intervals (95% CIs) for incident pre‐frailty and frailty with total choline and betaine. The proportional hazards assumption was tested using Schoenfeld residuals. Similar calculations were conducted to evaluate the relationships between lipid‐ and water‐soluble choline, plant‐ and animal‐derived choline and choline‐contributing compounds with incident pre‐frailty and frailty. Moreover, dose–response relationships were analysed through nonparametrically restricted cubic spline regression, with knots positioned at the 5th, 35th, 65th and 95th percentiles, exploring the association of choline with incident pre‐frailty and frailty.

Several covariates were adjusted for in the analysis. Model 1 did not adjust any confounders. In Model 2, age (years, continuous), sex (female or male), nationality (Han or minority), residence (urban or rural), geographical region (northeastern China, eastern China, central China, southern China or southwestern China), marital status (married, single, or divorced, widowed or separated), education (at or below primary school, middle school, or at or above high school), household per capita annual income (CNY, continuous), medical insurance (yes or no), drinking (never, moderate, or excess), smoking (never, previous or current), drinking water source (tap water, well water or other), cooking fuel type (clean fuel or polluting fuel), sedentary behaviour (hours/day, continuous) and dietary intake of energy (kcal/day, continuous), protein (g/day, continuous), fat (g/day, continuous) and carbohydrate (g/day, continuous). In Model 3, adjustments were additionally made for BMI (kg/m^2^, continuous), hypertension (yes or no), health infrastructure score (continuous), sanitation score (continuous) and social services score (continuous).

Additionally, we conducted several subgroup and interaction analyses to explore potential variations. These analyses were stratified by sex (male and female), age group (18–44, 45–59 and ≥ 60 years) and energy intake (tertiles). To assess the robustness of our findings, we conducted several sensitivity analyses. Firstly, we excluded participants who experienced an outcome event in the first 2 or 4 years of follow‐up to reduce potential reverse causality. Secondly, we excluded participants with (pre‐) frailty at baseline to confirm the stability of the frailty definition. Thirdly, to adjust for potential time‐varying confounding, we additionally adjusted for the survey year in the three models we constructed. Fourthly, to ensure the results were not influenced by variations in energy intake, we utilized energy adjustment models to calibrate the total choline intake [S10]. Finally, we further adjusted for lipid profiles to assess the impact of lipid levels on frailty; additional details regarding the analyses are presented in Supporting Information: Supplement Methods 3.

Analyses were conducted using R Version 4.3.1. All statistical tests were two‐tailed, and *p*‐values less than 0.05 were considered statistically significant.

## 3 Results

### 3.1 Baseline Characteristics

The baseline characteristics of participants stratified by quartiles of total choline intake are presented in Table 1. At baseline, the mean age was 46.4 years (SD 14.5), with 5420 (52.6%) being female. Participants with higher total choline intake were more likely to have elevated educational levels, higher income, increased BMI, a higher prevalence of hypertension, higher community index scores and greater intakes of energy, protein, fat and carbohydrate. Additionally, they were more likely to be currently married, engage in greater leisure sedentary time, reside in urban areas, never smoke, never drink, use tap water, have medical insurance, and utilize clean fuel. The mean (SD) intake of total choline and betaine was 245.3 (SD 212.0) mg/day and 113.5 (SD 102.8) mg/day, respectively. The intake of choline‐contributing compounds, including phosphatidylcholine, sphingomyelin, free choline, glycerophosphocholine and phosphocholine, was 144.0 (SD 161.3) mg/day, 9.8 (SD 7.7) mg/day, 64.2 (SD 48.6) mg/day, 20.4 (SD 17.2) mg/day and 7.0 (SD 4.2) mg/day, respectively. Participants displayed comparable baseline characteristics in the (pre‐) frailty analysis (Table S2).

**TABLE 1:** Baseline characteristics of participants by quartiles of total choline intake.

Characteristic	Total	Total choline intake quartiles			
		1	2	3	4
Participants, no. (%)	10 310 (100.0)	2578 (25.0)	2577 (25.0)	2577 (25.0)	2578 (25.0)
Age (years), mean (SD)	46.4 (14.5)	48.0 (15.4)	46.3 (14.2)	46.4 (14.1)	44.9 (13.9)
18–44, no. (%)	4815 (46.7)	1107 (42.9)	1218 (47.3)	1205 (46.8)	1285 (49.8)
45–59, no. (%)	3554 (34.5)	850 (33.0)	896 (34.7)	916 (35.5)	892 (34.6)
≥ 60, no. (%)	1941 (18.8)	621 (24.1)	463 (18.0)	456 (17.7)	401 (15.6)
Female, no. (%)	5420 (52.6)	1509 (58.5)	1434 (55.6)	1333 (51.7)	1144 (44.4)
Nationality, no. (%)					
Han	9139 (88.6)	2179 (84.5)	2283 (88.6)	2335 (90.6)	2342 (90.8)
Minority	1171 (11.4)	399 (15.5)	294 (11.4)	242 (9.4)	236 (9.2)
Residence, no. (%)					
Rural	6563 (63.7)	1992 (77.3)	1791 (69.5)	1559 (60.5)	1221 (47.4)
Urban	3747 (36.3)	586 (22.7)	786 (30.5)	1018 (39.5)	1357 (52.6)
Geographical region, no. (%)					
Northeastern China	2388 (23.2)	397 (15.4)	543 (21.1)	641 (24.9)	807 (31.3)
Eastern China	2600 (25.2)	429 (16.6)	590 (22.9)	755 (29.3)	826 (32.0)
Central China	1679 (16.3)	472 (18.3)	470 (18.2)	394 (15.3)	343 (13.3)
Southern China	2065 (20.0)	679 (26.3)	577 (22.4)	459 (17.8)	350 (13.6)
Southwestern China	1578 (15.3)	601 (23.3)	397 (15.4)	328 (12.7)	252 (9.8)
Marital status, no. (%)					
Married	8840 (85.7)	2172 (84.3)	2202 (85.4)	2224 (86.3)	2242 (87.0)
Never married	767 (7.4)	175 (6.8)	178 (6.9)	186 (7.2)	228 (8.8)
Divorced, widowed or separated	703 (6.8)	231 (9.0)	197 (7.6)	167 (6.5)	108 (4.2)
Education, no. (%)					
At or below primary school	3859 (37.4)	1392 (54.0)	1020 (39.6)	828 (32.1)	619 (24.0)
Middle school	4874 (47.3)	1041 (40.4)	1242 (48.2)	1289 (50.0)	1302 (50.5)
At or above high school	1577 (15.3)	145 (5.6)	315 (12.2)	460 (17.9)	657 (25.5)
Household per capita annual income (CNY), mean (SD)	9019.3 (12 418.7)	5229.6 (8867.8)	7964.0 (10 647.7)	10 482.2 (13 507.3)	12 401.5 (14 613.1)
Medical insurance, no. (%)	5045 (48.9)	877 (34.0)	1220 (47.3)	1385 (53.7)	1563 (60.6)
Drinking, no. (%)					
Never	7366 (71.4)	2030 (78.7)	1887 (73.2)	1791 (69.5)	1658 (64.3)
Moderate	2269 (22.0)	431 (16.7)	546 (21.2)	601 (23.3)	691 (26.8)
Excess	675 (6.5)	117 (4.5)	144 (5.6)	185 (7.2)	229 (8.9)
Smoking, no. (%)					
Never	7051 (68.4)	1831 (71.0)	1822 (70.7)	1716 (66.6)	1682 (65.2)
Previous	352 (3.4)	77 (3.0)	78 (3.0)	93 (3.6)	104 (4.0)
Current	2907 (28.2)	670 (26.0)	677 (26.3)	768 (29.8)	792 (30.7)
Drinking water source, no. (%)					
Tap water	8186 (79.4)	1758 (68.2)	2056 (79.8)	2128 (82.6)	2244 (87.0)
Well water	1844 (17.9)	685 (26.6)	461 (17.9)	401 (15.6)	297 (11.5)
Other	280 (2.7)	135 (5.2)	60 (2.3)	48 (1.9)	37 (1.4)
Cooking fuel type, no. (%)					
Clean fuel	5787 (56.1)	975 (37.8)	1374 (53.3)	1582 (61.4)	1856 (72.0)
Polluting fuel	4523 (43.9)	1603 (62.2)	1203 (46.7)	995 (38.6)	722 (28.0)
Sedentary behaviour (hours/day), mean (SD)	1.8 (1.5)	1.7 (1.6)	1.8 (1.4)	1.8 (1.5)	1.8 (1.5)
BMI (kg/m^2^), mean (SD)	23.2 (3.4)	22.7 (3.5)	23.2 (3.3)	23.4 (3.4)	23.6 (3.5)
SBP (mmHg), mean (SD)	121.0 (16.4)	121.2 (17.5)	120.4 (16.1)	121.4 (16.9)	120.9 (15.2)
DBP (mmHg), mean (SD)	78.2 (10.3)	78.0 (10.9)	77.8 (10.3)	78.5 (10.1)	78.6 (10.0)
Hypertension, no. (%)	5659 (54.9)	1388 (53.8)	1396 (54.2)	1445 (56.1)	1430 (55.5)
Dietary intake					
Energy (kcal/day), mean (SD)	2085.0 (657.9)	1842.4 (579.7)	2002.3 (619.4)	2130.9 (647.7)	2364.2 (671.2)
Protein (g/day), mean (SD)	65.8 (24.0)	49.8 (15.5)	60.7 (18.6)	69.1 (21.3)	83.6 (25.5)
Fat (g/day), mean (SD)	70.4 (37.3)	52.7 (30.1)	65.6 (34.1)	74.8 (35.6)	88.5 (39.4)
Carbohydrate (g/day), mean (SD)	293.9 (114.1)	291.2 (109.9)	290.0 (116.5)	291.6 (117.2)	302.7 (112.1)
Community index					
Health infrastructure score, mean (SD)	5.5 (2.4)	4.9 (2.3)	5.5 (2.3)	5.5 (2.4)	5.9 (2.4)
Sanitation score, mean (SD)	6.9 (2.9)	5.9 (3.0)	6.8 (2.9)	7.3 (2.8)	7.8 (2.6)
Social services score, mean (SD)	3.6 (3.1)	2.9 (2.7)	3.5 (3.0)	3.8 (3.1)	4.2 (3.2)
Total choline (mg/day), mean (SD)	245.3 (212.0)	96.1 (26.7)	171.0 (20.2)	249.0 (26.0)	465.0 (319.2)
Lipid‐soluble choline^a^ (mg/day), mean (SD)	153.8 (166.2)	45.2 (21.7)	98.8 (27.4)	156.0 (38.1)	314.8 (259.0)
Water‐soluble choline^b^ (mg/day), mean (SD)	91.6 (61.9)	52.6 (16.9)	73.7 (23.4)	94.0 (32.6)	145.9 (93.0)
Phosphatidylcholine (mg/day), mean (SD)	144.0 (161.3)	41.3 (19.9)	91.2 (25.5)	145.5 (36.0)	297.9 (254.4)
Sphingomyelin (mg/day), mean (SD)	9.8 (7.7)	3.9 (3.3)	7.6 (4.3)	10.9 (5.4)	16.9 (9.2)
Free choline (mg/day), mean (SD)	64.2 (48.6)	36.7 (14.3)	51.8 (21.2)	65.9 (29.5)	102.3 (74.5)
Glycerophosphocholine (mg/day), mean (SD)	20.4 (17.2)	11.0 (4.5)	15.7 (6.4)	20.5 (9.2)	34.4 (27.0)
Phosphocholine (mg/day), mean (SD)	7.0 (4.2)	5.0 (2.9)	6.2 (3.3)	7.5 (3.9)	9.2 (5.2)
Betaine (mg/day), mean (SD)	113.5 (102.8)	102.0 (101.9)	109.4 (103.4)	116.3 (97.3)	126.2 (106.7)

*Note:* The mean of three measurements of systolic and diastolic blood pressure was used.

Abbreviations: BMI, body mass index; DBP, diastolic blood pressure; SBP, systolic blood pressure.

^a^ Lipid‐soluble choline includes phosphatidylcholine and sphingomyelin.

^b^ Water‐soluble choline includes free choline, glycerophosphocholine and phosphocholine.

### 3.2 Dietary Choline and Betaine Intake With Pre‐Frailty and Frailty

Over a median of 6.1 years (P_25_–P_75_, 4.0–11.0 years) follow‐up, 1150 cases (11.2%) of incident frailty were recorded for the analysis of frailty. During a median follow‐up duration of 5.0 years (P_25_–P_75_, 3.1–9.0 years), 2376 cases (34.1%) of incident pre‐frailty were documented. For the analysis of (pre‐) frailty, across a median follow‐up period of 5.0 years (P_25_–P_75_, 3.0–9.0 years), 2629 cases (36.4%) of incident (pre‐) frailty were recorded (Figure S1).

**FIGURE 1:**
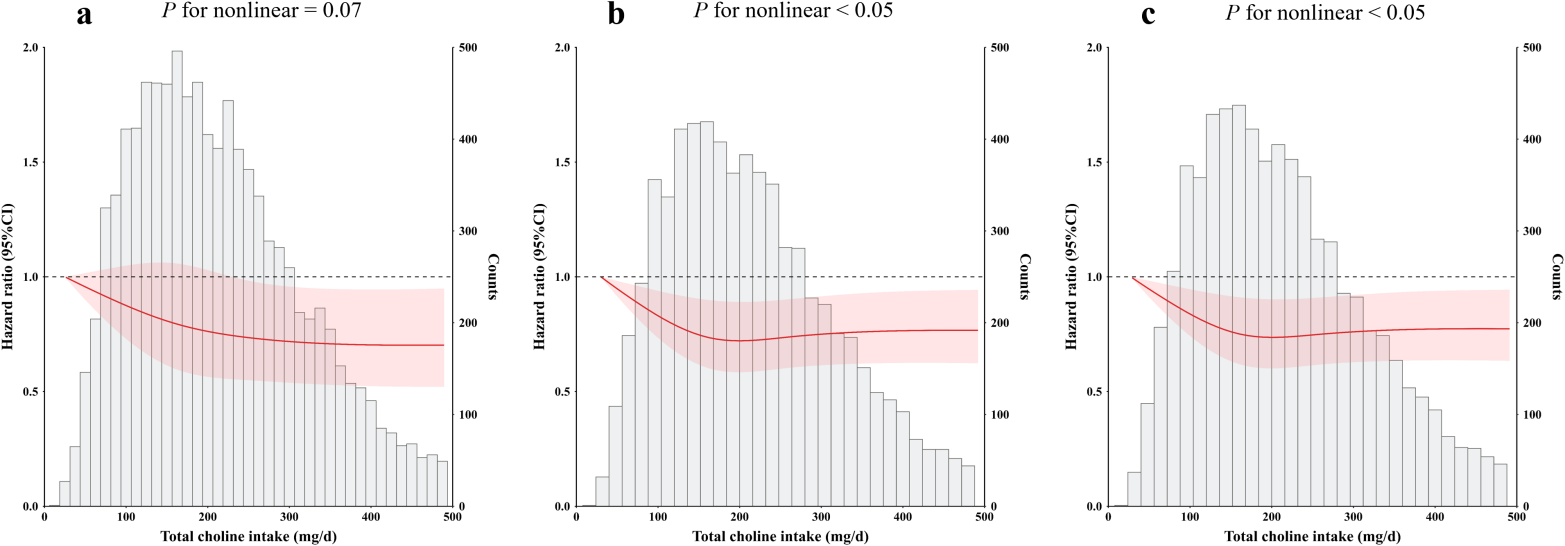
Dose‐respond associations of total choline intake with incident pre‐frailty and frailty. (a: associations of total choline intake with incident frailty; b: associations of total choline intake with incident pre‐frailty; c: associations of total choline intake with incident (pre‐) frailty). The term (pre‐) frailty refers to the combined states of pre‐frailty and frailty. Adjusted for age (years, continuous), sex (female or male), nationality (Han or minority), residence (urban or rural), geographical region (northeastern China, eastern China, central China, southern China or southwestern China), marital status (married, single, or divorced, widowed or separated), education (at or below primary school, middle school, or at or above high school), household per capita annual income (CNY, continuous), medical insurance (yes or no), drinking (never, moderate or excess), smoking (never, previous or current), drinking water source (tap water, well water or other), cooking fuel type (clean fuel fuel or polluting fuel), sedentary behaviour (hours/day, continuous), dietary intake of energy (kcal/day, continuous), protein (g/day, continuous), fat (g/day, continuous) and carbohydrate (g/day, continuous), body mass index (kg/m^2^, continuous), hypertension (yes or no), health infrastructure score (continuous), sanitation score (continuous) and social services score (continuous).

Cox models with penalized splines showed statistically significant L‐shaped associations for total choline with pre‐frailty and frailty (Figure 1). Table 2 presents the association of total choline intake with incident pre‐frailty and frailty. Categorizing total choline intake into quartiles, compared with the 1st quartile, those in the 2nd to 4th quartiles had lower frailty risks with HRs of 0.84 (95% CI: 0.71, 0.98), 0.80 (95% CI: 0.67, 0.95) and 0.75 (95% CI: 0.61, 0.93), respectively. Compared with those in the 1st quartile of total choline, participants in the 3rd quartile had lower odds, with HRs of 0.87 (95% CI: 0.77, 0.99) and 0.85 (95% CI: 0.76, 0.96) for pre‐frailty and (pre‐) frailty, respectively.

**TABLE 2:** Associations of total choline intake with incident pre‐frailty and frailty.

Outcomes	Total choline intake quartiles			
	1	2	3	4
Frailty (*n* = 10 310)				
Mean intake (SD), mg/day	96.14 (26.72)	171.00 (20.24)	249.03 (25.97)	464.99 (319.20)
Events/participants	368/2578	276/2577	264/2577	242/2578
Incidence rate per 1000 person‐years	19.70	15.77	15.61	14.82
Model 1^a^, HR (95% CI)	1 (reference)	0.81 (0.69, 0.95)	0.80 (0.68, 0.94)	0.75 (0.64, 0.88)
Model 2^b^, HR (95% CI)	1 (reference)	0.84 (0.72, 0.99)	0.79 (0.66, 0.94)	0.75 (0.61, 0.92)
Model 3^c^, HR (95% CI)	1 (reference)	0.84 (0.71, 0.98)	0.80 (0.67, 0.95)	0.75 (0.61, 0.93)
Pre‐frailty (*n* = 6969)				
Mean intake (SD), mg/day	98.64 (27.06)	172.90 (19.98)	250.09 (25.96)	460.63 (250.90)
Events/participants	666/1743	597/1742	550/1742	563/1742
Incidence rate per 1000 person‐years	61.92	58.25	54.61	58.44
Model 1^a^, HR (95% CI)	1 (reference)	0.94 (0.84, 1.05)	0.88 (0.79, 0.98)	0.93 (0.83, 1.04)
Model 2^b^, HR (95% CI)	1 (reference)	0.97 (0.87, 1.09)	0.87 (0.77, 0.99)	0.98 (0.85, 1.13)
Model 3^c^, HR (95% CI)	1 (reference)	0.96 (0.86, 1.08)	0.87 (0.77, 0.99)	0.97 (0.84, 1.12)
(Pre‐) frailty^d^ (*n* = 7222)				
Mean intake (SD), mg/day	98.07 (26.93)	171.94 (19.91)	249.19 (26.01)	459.43 (248.22)
Events/participants	748/1806	659/1805	603/1805	619/1806
Incidence rate per 1000 person‐years	68.09	62.70	58.26	62.51
Model 1^a^, HR (95% CI)	1 (reference)	0.92 (0.83, 1.02)	0.85 (0.76, 0.95)	0.91 (0.82, 1.01)
Model 2^b^, HR (95% CI)	1 (reference)	0.96 (0.86, 1.07)	0.85 (0.76, 0.96)	0.96 (0.84, 1.10)
Model 3^c^, HR (95% CI)	1 (reference)	0.95 (0.85, 1.06)	0.85 (0.76, 0.96)	0.95 (0.83, 1.09)

^a^ The crude model was not adjusted for any confounders.

^b^ Adjusted for age (years, continuous), sex (female or male), nationality (Han or minority), residence (urban or rural), geographical region (northeastern China, eastern China, central China, southern China or southwestern China), marital status (married, single, or divorced, widowed or separated), education (at or below primary school, middle school, or at or above high school), household per capita annual income (CNY, continuous), medical insurance (yes or no), drinking (never, moderate or excess), smoking (never, previous or current), drinking water source (tap water, well water or other), cooking fuel type (clean fuel or polluting fuel), sedentary behaviour (hours/day, continuous), dietary intake of energy (kcal/day, continuous), protein (g/day, continuous), fat (g/day, continuous) and carbohydrate (g/day, continuous).

^c^ Additionally adjusted for body mass index (kg/m^2^, continuous), hypertension (yes or no), health infrastructure score (continuous), sanitation score (continuous) and social services score (continuous).

^d^ The term (pre‐) frailty refers to the combined states of pre‐frailty and frailty.

**FIGURE 2:**
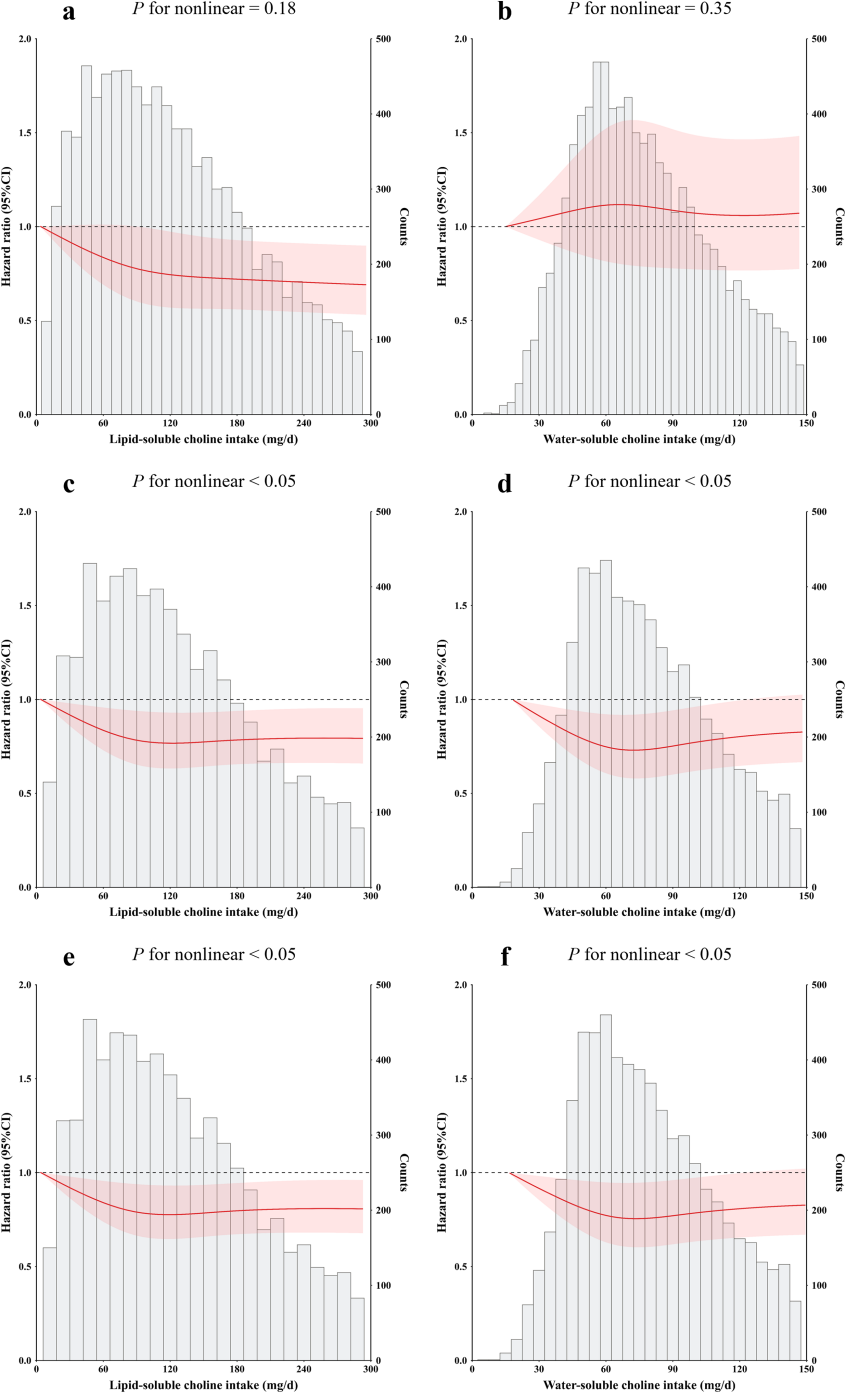
Dose‐respond associations of lipid‐ and water‐soluble choline intake with incident pre‐frailty and frailty. (a, b: associations of lipid‐ and water‐soluble choline intake with incident frailty; c, d: associations of lipid‐ and water‐soluble choline intake with incident pre‐frailty; e, f: associations of lipid‐ and water‐soluble intake choline with incident (pre‐) frailty). The term (pre‐) frailty refers to the combined states of pre‐frailty and frailty. Adjusted for age (years, continuous), sex (female or male), nationality (Han or minority), residence (urban or rural), geographical region (northeastern China, eastern China, central China, southern China or southwestern China), marital status (married, single, or divorced, widowed or separated), education (at or below primary school, middle school, or at or above high school), household per capita annual income (CNY, continuous), medical insurance (yes or no), drinking (never, moderate or excess), smoking (never, previous or current), drinking water source (tap water, well water or other), cooking fuel type (clean fuel fuel or polluting fuel), sedentary behaviour (hours/day, continuous), dietary intake of energy (kcal/day, continuous), protein (g/day, continuous), fat (g/day, continuous) and carbohydrate (g/day, continuous), body mass index (kg/m^2^, continuous), hypertension (yes or no), health infrastructure score (continuous), sanitation score (continuous) and social services score (continuous).

A negative dose–response association of lipid‐ and water‐soluble choline intake with incident pre‐frailty and frailty is shown in Figure 2. Participants in the 2nd to 4th quartiles of lipid‐soluble choline exhibited 18% (HR: 0.82; 95% CI: 0.69, 0.98) to 23% (HR: 0.77; 95% CI: 0.63, 0.95) lower risks compared with the 1st quartile. Participants in the 3rd quartile of lipid‐soluble choline had lower pre‐frailty and (pre‐) frailty risks with HRs of 0.88 (95% CI: 0.77, 0.99) and 0.87 (95% CI: 0.77, 0.98) compared with the 1st quartile, respectively. Participants in the 2nd quartile of water‐soluble choline intake demonstrated lower odds compared with those in the 1st quartile, with HRs of 0.83 (95% CI: 0.74, 0.94) and 0.86 (95% CI: 0.77, 0.97) for pre‐frailty and (pre‐) frailty, respectively (Table S3).

Subsequent analysis examined the relationship of choline‐contributing compounds with pre‐frailty and frailty. Consumption in the 3rd to 4th quartiles of phosphatidylcholine was associated with a 19% (HR: 0.81; 95% CI: 0.68, 0.96) to 23% (HR: 0.77; 95% CI: 0.63, 0.94) reduction in frailty risk compared with the 1st quartile (Table 3, Figure S2). Our analysis indicated that consumption in the 3rd quartile of phosphatidylcholine (HR: 0.84; 95% CI: 0.74, 0.95) and sphingomyelin (HR: 0.84; 95% CI: 0.74, 0.96) was associated with a 16% reduction in the odds of pre‐frailty compared with the 1st quartile (Table S4). Further investigation into the (pre‐) frailty showed that both phosphatidylcholine and sphingomyelin intake significantly reduced (pre‐) frailty risk, with HRs of 0.84 (95% CI: 0.75, 0.95) and 0.84 (95% CI: 0.75, 0.96), respectively (Table S5). Participants in the 2nd quartile of betaine had obviously lower odds of incident pre‐frailty, with an HR of 0.86 (95% CI: 0.76, 0.97) (Table S4).

**TABLE 3:** Associations of choline‐contributing compounds and betaine intake with incident frailty.

Dietary choline and betaine	Choline‐contributing compounds and betaine intake quartiles			
	1	2	3	4
Phosphatidylcholine				
Mean intake (SD), mg/day	38.17 (15.58)	87.72 (14.44)	145.20 (19.94)	304.93 (250.90)
Events/participants	365/2578	279/2577	257/2577	249/2578
Model 1^a^, HR (95% CI)	1 (reference)	0.86 (0.74, 1.01)	0.82 (0.70, 0.96)	0.84 (0.71, 0.99)
Model 2^b^, HR (95% CI)	1 (reference)	0.83 (0.71, 0.98)	0.80 (0.67, 0.96)	0.75 (0.61, 0.92)
Model 3^c^, HR (95% CI)	1 (reference)	0.85 (0.73, 1.01)	0.81 (0.68, 0.96)	0.77 (0.63, 0.94)
Sphingomyelin				
Mean intake (SD), mg/day	1.99 (1.51)	6.57 (1.15)	10.90 (1.43)	19.89 (7.41)
Events/participants	360/2580	294/2575	270/2577	226/2578
Model 1^a^, HR (95% CI)	1 (reference)	0.94 (0.81, 1.10)	0.94 (0.81, 1.11)	0.90 (0.76, 1.07)
Model 2^b^, HR (95% CI)	1 (reference)	0.93 (0.79, 1.10)	0.92 (0.76, 1.10)	0.91 (0.72, 1.16)
Model 3^c^, HR (95% CI)	1 (reference)	0.96 (0.81, 1.13)	0.95 (0.79, 1.14)	0.93 (0.73, 1.18)
Free choline				
Mean intake (SD), mg/d	27.48 (6.49)	44.27 (4.65)	63.73 (6.94)	121.21 (65.90)
Events/participants	300/2578	297/2577	273/2577	280/2578
Model 1^a^, HR (95% CI)	1 (reference)	0.98 (0.83, 1.15)	0.87 (0.74, 1.03)	0.81 (0.69, 0.95)
Model 2^b^, HR (95% CI)	1 (reference)	1.03 (0.87, 1.22)	0.97 (0.81, 1.16)	1.01 (0.83, 1.22)
Model 3^c^, HR (95% CI)	1 (reference)	1.08 (0.91, 1.27)	0.99 (0.82, 1.19)	1.02 (0.84, 1.23)
Glycerophosphocholine				
Mean intake (SD), mg/day	8.49 (1.89)	13.55 (1.41)	19.71 (2.33)	39.87 (24.53)
Events/participants	354/2578	277/2577	272/2577	247/2578
Model 1^a^, HR (95% CI)	1 (reference)	0.80 (0.69, 0.94)	0.79 (0.67, 0.92)	0.80 (0.68, 0.94)
Model 2^b^, HR (95% CI)	1 (reference)	0.96 (0.82, 1.14)	1.03 (0.86, 1.23)	1.05 (0.85, 1.29)
Model 3^c^, HR (95% CI)	1 (reference)	0.96 (0.81, 1.13)	1.00 (0.83, 1.19)	1.10 (0.89, 1.35)
Phosphocholine				
Mean intake (SD), mg/day	2.85 (0.86)	5.13 (0.60)	7.38 (0.76)	12.53 (4.27)
Events/participants	321/2578	277/2577	290/2577	262/2578
Model 1^a^, HR (95% CI)	1 (reference)	0.93 (0.79, 1.09)	1.04 (0.88, 1.22)	0.99 (0.84, 1.17)
Model 2^b^, HR (95% CI)	1 (reference)	1.06 (0.90, 1.26)	1.18 (0.99, 1.40)	1.07 (0.89, 1.29)
Model 3^c^, HR (95% CI)	1 (reference)	1.06 (0.90, 1.25)	1.20 (1.00, 1.42)	1.14 (0.95, 1.38)
Betaine				
Mean intake (SD), mg/day	20.24 (12.66)	66.50 (12.40)	115.94 (17.54)	251.19 (108.38)
Events/participants	301/2578	289/2577	278/2577	282/2578
Model 1^a^, HR (95% CI)	1 (reference)	1.03 (0.87, 1.21)	1.02 (0.86, 1.20)	0.97 (0.82, 1.14)
Model 2^b^, HR (95% CI)	1 (reference)	1.05 (0.88, 1.24)	1.12 (0.94, 1.33)	1.09 (0.91, 1.31)
Model 3^c^, HR (95% CI)	1 (reference)	0.99 (0.83, 1.17)	1.05 (0.88, 1.25)	0.98 (0.81, 1.18)

^a^ The crude model was not adjusted for any confounders.

^b^ Adjusted for age (years, continuous), sex (female or male), nationality (Han or minority), residence (urban or rural), geographical region (northeastern China, eastern China, central China, southern China or southwestern China), marital status (married, single, or divorced, widowed or separated), education (at or below primary school, middle school, or at or above high school), household per capita annual income (CNY, continuous), medical insurance (yes or no), drinking (never, moderate or excess), smoking (never, previous or current), drinking water source (tap water, well water or other), cooking fuel type (clean fuel or polluting fuel), sedentary behaviour (hours/day, continuous), dietary intake of energy (kcal/day, continuous), protein (g/day, continuous), fat (g/day, continuous) and carbohydrate (g/day, continuous).

^c^ Additionally adjusted for body mass index (kg/m^2^, continuous), hypertension (yes or no), health infrastructure score (continuous), sanitation score (continuous) and social services score (continuous).

We also examined the association of choline intake from different food sources with incident pre‐frailty and frailty. We found that major food groups providing choline were eggs (63.71 mg/day) and vegetables and fruits (50.75 mg/day) (Figure S3). Consumption in the 3rd quartiles of choline from plant‐derived sources had lower frailty risks with HRs of 0.83 (95% CI: 0.69, 0.99) compared with the 1st quartile (Table S6). No statistically significant association was observed between choline from animal‐derived sources and incident pre‐frailty or frailty.

### 3.3 Subgroups and Sensitivity Analyses

We performed subgroup analyses according to sex, age and energy intake (Tables S7–S9). Notably, we observed that energy intake exhibits an interaction effect on (pre‐) frailty (*p* = 0.0377) (Table S9). The sensitivity analyses for frailty were consistent with primary findings after adjusting for multiple variables (Table S10). The findings persisted with additional adjustments for survey years in each model. Additionally, the associations of total choline with frailty were further reinforced by excluding participants within the first 2 and 4 years of follow‐up or with (pre‐) frailty at baseline (Table S10). The HRs (95% CI) only had minor variations after the energy adjustment model calibrated the total choline intake for frailty (Table S10). Moreover, further adjustment for lipid profiles yielded only minimal changes in the outcomes (Table S10).

## 4 Discussion

In this prospective, household‐based cohort study using data from CHNS, we observed L‐shaped associations between total choline intake and the odds of incident pre‐frailty and frailty. Analysis of choline‐contributing compounds revealed that phosphatidylcholine intake was negatively associated with increased odds of pre‐frailty and frailty, while sphingomyelin intake was associated with pre‐frailty and combined (pre‐) frailty. Betaine intake was found to be linked to reduced odds of pre‐frailty. Furthermore, lipid‐soluble choline intake was associated with lower odds of incident pre‐frailty and frailty, whereas moderate intake of water‐soluble choline demonstrated associations with pre‐frailty and (pre‐) frailty. Notably, plant‐derived sources of choline were significantly associated with a reduced odds of incident frailty. These findings provide epidemiological evidence supporting dietary strategies for the prevention and management of frailty in adults, underscoring its public health significance.

Choline plays a pivotal role in numerous fundamental physiological functions throughout the human life cycle and is recognized as an essential dietary nutrient [25]. Previous studies have demonstrated an inverse association between choline intake and adverse health‐related outcomes, including cardiovascular disease, osteoporosis and other conditions [26, 27]. Lee et al. aimed to examine the relationship between total dietary choline intake and muscle responses to resistance exercise training (RET) in older adults with healthy conditions (*n* = 46) [28]. They subsequently conducted a randomized controlled trial (*n* = 37) and verified that low total choline intake may negatively affect strength gains with RET [29]. Mone et al. conducted a randomized controlled trial (*n* = 99) focusing on cognitive frailty in hypertensive older adults, examining the effects of choline bitartrate combined with vitamin B_12_ on cognitive impairment [30]. These studies offer preliminary insights into the relationship between choline and frailty, but they are limited by small sample sizes and short‐term designs. Our research extends this body of knowledge by developing a FI, primarily based on the principle of cumulative deficits, which includes 27 items to reflect overall health status rather than focusing on individual dimensions such as muscle strength or cognition. Additionally, we conducted a comprehensive cohort study in Chinese adults, examining the association between dietary choline, its derivatives, soluble forms, food sources and frailty. Our findings indicated that participants with a moderate to high intake of total choline showed a lower risk of incident frailty. Furthermore, our results remained statistically significant after excluding participants who experienced an outcome within the first 4 years of follow‐up. The outcomes of our study substantiate the viewpoint of choline as an independent preventive factor for frailty.

We also investigated the relationship between the intake of different forms of soluble choline and its subtypes with incident frailty. Our study demonstrated that the consumption of lipid‐soluble choline is significantly associated with a reduced risk of frailty, and phosphatidylcholine is associated with a decreased risk of frailty. As a primary source of dietary choline, phosphatidylcholine has been shown in an RCT study to improve muscle health, mainly due to its role in maintaining cell membrane integrity and promoting muscle function [S11]. Phosphatidylcholine serves as a precursor to phosphatidic acid, which is crucial for activating mammalian target of rapamycin (mTOR), a key regulator of protein synthesis that promotes muscle growth and repair. Thus, adequate intake of phosphatidylcholine may indirectly enhance muscle quality [S12]. Additionally, prior studies of metabolomic profile and dietary patterns have shown that phosphatidylcholine is significantly associated with the Mediterranean dietary pattern (MD) [31]. One meta‐analysis has indicated that adherence to MD could significantly mitigate the risk of frailty [32].

Interestingly, we found that plant‐derived choline, but not animal‐derived choline, significantly reduced the odds of frailty. Our results indicated that the primary dietary sources of plant‐derived choline include vegetables, fruits, potatoes, beans, nuts and grains. Plant‐derived diets are rich in diverse nutrients that may synergize with choline to enhance its protective effects against frailty. Thus, the inverse association observed may partly reflect contributions from other beneficial components in these foods. Choline may influence protein homeostasis by increasing synthesis and reducing breakdown. Evidence suggests a negative association between plant protein intake and frailty, highlighting the potential synergistic effects of plant‐derived choline and protein in enhancing muscle health [33]. A cohort study among Chinese elderly reported that a healthful plant‐derived diet was associated with decreased frailty risk [10], consistent with findings from a study of elderly American women [34]. These findings support the role of plant‐derived diets in frailty prevention and suggest that the protective effects of choline may be influenced by its specific form and dietary source rather than choline alone. Future studies should explore the metabolic mechanisms of different choline types and sources and their associations with frailty.

Frailty is an age‐related syndrome characterized by a decline in multiple system functions, leading to physical weakness, reduced mobility and decreased resistance to disease [35]. The potential protective role of choline against frailty may be attributed to several mechanisms. Firstly, choline is essential for muscle health as it serves as a precursor to acetylcholine, a critical neurotransmitter for muscle signal transmission [36]. A deficiency in choline may impair muscle function and strength, thereby contributing to the development of frailty [37]. Secondly, choline aids in lipid metabolism and the prevention of hepatic steatosis. The accumulation of fat in the liver and the presence of metabolic disorders have been associated with the onset of frailty [38]. Thirdly, choline is involved in modulating inflammatory responses within the body. Frailty is often accompanied by chronic low‐grade inflammation, and sufficient choline intake may help regulate the immune function and reduce inflammation, thereby mitigating frailty symptoms [39]. Additionally, choline acts as a methyl donor and participates in various methylation reactions that are crucial for DNA repair, gene expression and detoxification processes [40]. Poor methylation can lead to a variety of health issues, including frailty.

### 4.1 Strengths and Limitations

This study has several strengths, including its prospective design, large sample size, and comprehensive range of choline intake data. The detailed information enabled us to examine choline derivatives, different soluble forms and food sources, which are often overlooked in other studies.

There are also several limitations in this study. Firstly, dietary choline intake is assessed using three consecutive 24h‐DRs, which are susceptible to measurement error and may not accurately reflect long‐term choline intake. However, we incorporated two weekdays and one weekend day to investigate dietary patterns that closely approximate the habitual intake of the study participants. Secondly, our study outcome is primarily concerned with the incident frailty. However, given that frailty is a dynamic and modifiable process, further research could explore the association between choline and changes in frailty status. Thirdly, similar to other traditional observational cohort studies, despite our adjustment for multiple confounders, the potential for confounding bias might remain, such as genetic susceptibility. Fourthly, our study only focused on the Chinese population, which may restrict the generalizability of findings to other ethnic and racial demographics. Lastly, lipid profiles were only collected in the 2009 survey wave (Table S11), limiting their use as a covariate throughout the study period. However, sensitivity analyses using imputed lipid data yielded results consistent with the main analysis, while findings from the 2009 dataset showed slight attenuation.

## 5 Conclusions

In conclusion, moderate to high dietary choline intake (171.00–464.99 mg/day), particularly phosphatidylcholine (145.20–304.93 mg/day), may be associated with reduced odds of frailty.

## Conflicts of Interest

The authors declare no conflicts of interest.

## Supporting information


**Figure S1.** Flowchart of recruitment and follow‐up.
**Figure S2.** Dose‐respond associations of choline‐contributing compounds and betaine intake with incident frailty.
**Figure S3.** Distribution of total choline intake from various food sources.
**Table S1.** The 27 items and cut‐points for the frailty index.
**Table S2.** Baseline characteristics of participants by quartiles of total choline intake.
**Table S3.** Associations of lipid‐ and water‐soluble choline intake with incident pre‐frailty and frailty.
**Table S4.** Associations of choline‐contributing compounds and betaine intake with incident pre‐frailty.
**Table S5.** Associations of choline‐contributing compounds and betaine intake with incident (pre‐) frailty.
**Table S6.** Associations of choline intake from different food sources with incident pre‐frailty and frailty.
**Table S7.** Associations of total choline intake with incident frailty: subgroup analysis stratified by sex, age and energy intake.
**Table S8.** Associations of total choline with incident pre‐frailty: subgroup analysis stratified by sex, age and energy intake.
**Table S9.** Associations of total choline intake with incident (pre‐) frailty: subgroup analysis stratified by sex, age and energy intake.
**Table S10.** Associations of total choline intake with incident frailty: sensitivity analysis.
**Table S11.** Characteristics of participants for lipid profiles.

## Data Availability

Study protocol: Not available. Statistical code: Not available. Data set: Available from the China Health and Nutrition Survey on request (https://www.cpc.unc.edu/projects/china).

## References

[1] C. Casals , L. Ávila‐Cabeza‐de‐Vaca , A. González‐Mariscal , et al., “Effects of an Educational Intervention on Frailty Status, Physical Function, Physical Activity, Sleep Patterns, and Nutritional Status of Older Adults With Frailty or Pre‐Frailty: The FRAGSALUD Study,” Frontiers in Public Health 11 (2023): 1267666. 38098822 10.3389/fpubh.2023.1267666 PMC10720710

[2] R. O'Caoimh , D. Sezgin , M. R. O'Donovan , et al., “Prevalence of Frailty in 62 Countries Across the World: A Systematic Review and Meta‐Analysis of Population‐Level Studies,” Age and Ageing 50, no. 1 (2021): 96–104. 33068107 10.1093/ageing/afaa219

[3] E. Dent , F. C. Martin , H. Bergman , J. Woo , R. Romero‐Ortuno , and J. D. Walston , “Management of Frailty: Opportunities, Challenges, and Future Directions,” Lancet 394, no. 10206 (2019): 1376–1386. 31609229 10.1016/S0140-6736(19)31785-4

[4] W. Li , D. Chen , W. Ruan , Y. Peng , Z. Lu , and D. Wang , “Associations of Depression, Sleep Disorder With Total and Cause‐Specific Mortality: A Prospective Cohort Study,” Journal of Affective Disorders 298, no. Pt A (2022): 134–141. 34763200 10.1016/j.jad.2021.10.131

[5] C. de Labra , C. Guimaraes‐Pinheiro , A. Maseda , T. Lorenzo , and J. C. Millán‐Calenti , “Effects of Physical Exercise Interventions in Frail Older Adults: A Systematic Review of Randomized Controlled Trials,” BMC Geriatrics 15 (2015): 154. 26626157 10.1186/s12877-015-0155-4 PMC4667405

[6] H. J. Coelho‐Junior , E. Marzetti , A. Picca , M. Cesari , M. C. Uchida , and R. Calvani , “Protein Intake and Frailty: A Matter of Quantity, Quality, and Timing,” Nutrients 12, no. 10 (2020): 2915. 32977714 10.3390/nu12102915 PMC7598653

[7] U. Kansakar , V. Trimarco , P. Mone , F. Varzideh , A. Lombardi , and G. Santulli , “Choline Supplements: An Update,” Frontiers in Endocrinology 14 (2023): 1148166. 36950691 10.3389/fendo.2023.1148166 PMC10025538

[8] J. Yuan , X. Liu , C. Liu , et al., “Is Dietary Choline Intake Related to Dementia and Alzheimer's Disease Risks? Results From the Framingham Heart Study,” American Journal of Clinical Nutrition 116, no. 5 (2022): 1201–1207. 37208066 10.1093/ajcn/nqac193 PMC9630864

[9] M. Golzarand , Z. Bahadoran , P. Mirmiran , and F. Azizi , “Dietary Choline and Betaine Intake and Risk of Hypertension Development: A 7.4‐Year Follow‐Up,” Food & Function 12, no. 9 (2021): 4072–4078. 33977970 10.1039/d0fo03208e

[10] R. Qi , Y. Yang , B. Sheng , H. Li , and X. Zhang , “Plant‐Based Diet Indices and Their Association With Frailty in Older Adults: A CLHLS‐Based Cohort Study,” Nutrients 15, no. 24 (2023): 5120. 38140379 10.3390/nu15245120 PMC10745508

[11] M. Yan , Y. Liu , L. Wu , et al., “The Association Between Dietary Purine Intake and Mortality: Evidence From the CHNS Cohort Study,” Nutrients 14, no. 9 (2022): 1718. 35565687 10.3390/nu14091718 PMC9102343

[12] F. Zhai , X. Guo , B. M. Popkin , et al., “Evaluation of the 24‐Hour Individual Recall Method in China,” Food and Nutrition Bulletin 17, no. 2 (1996): 1–7.

[13] K. B. S. Patterson , J. Williams , J. Howe , and J. Holden , “USDA Database for the Choline Content of Common Foods,” Release Two, 2008, http://www.ars.usda.gov/SP2UserFiles/Place/80400525/Data/Choline/Choln02.pdf.

[14] S. H. Zeisel , M. H. Mar , J. C. Howe , and J. M. Holden , “Concentrations of Choline‐Containing Compounds and Betaine in Common Foods,” Journal of Nutrition 133, no. 5 (2003): 1302–1307. 12730414 10.1093/jn/133.5.1302

[15] H. J. Yang , Y. Kang , Y. Z. Li , et al., “Relationship Between Different Forms of Dietary Choline and Ovarian Cancer Survival: Findings From the Ovarian Cancer Follow‐Up Study, a Prospective Cohort Study,” Food & Function 13, no. 23 (2022): 12342–12352. 36354156 10.1039/d2fo02594a

[16] A. B. Mitnitski , A. J. Mogilner , and K. Rockwood , “Accumulation of Deficits as a Proxy Measure of Aging,” Scientific World Journal 1 (2001): 323–336. 12806071 10.1100/tsw.2001.58 PMC6084020

[17] K. Rockwood , X. Song , C. MacKnight , et al., “A Global Clinical Measure of Fitness and Frailty in Elderly People,” CMAJ 173, no. 5 (2005): 489–495. 16129869 10.1503/cmaj.050051 PMC1188185

[18] D. He , Z. Wang , J. Li , et al., “Changes in Frailty and Incident Cardiovascular Disease in Three Prospective Cohorts,” European Heart Journal 45, no. 12 (2024): 1058–1068. 38241094 10.1093/eurheartj/ehad885

[19] J. N. Fan , Z. J. Sun , C. Q. Yu , et al., “Comparison of Fried Phenotype and Frailty Index and Their Associations With Risk of Mortality,” Zhonghua Liu Xing Bing Xue Za Zhi 42, no. 7 (2021): 1179–1187. 34814528 10.3760/cma.j.cn112338-20210310-00192

[20] X. M. Wang , W. F. Zhong , Z. H. Li , et al., “Dietary Diversity and Frailty Among Older Chinese People: Evidence From the Chinese Longitudinal Healthy Longevity Study,” American Journal of Clinical Nutrition 117, no. 2 (2023): 383–391. 36811562 10.1016/j.ajcnut.2022.11.017

[21] K. Jayanama , O. Theou , J. M. Blodgett , L. Cahill , and K. Rockwood , “Frailty, Nutrition‐Related Parameters, and Mortality Across the Adult Age Spectrum,” BMC Medicine 16, no. 1 (2018): 188. 30360759 10.1186/s12916-018-1176-6 PMC6202862

[22] J. L. Atkins , J. Jylhävä , N. L. Pedersen , et al., “A Genome‐Wide Association Study of the Frailty Index Highlights Brain Pathways in Ageing,” Aging Cell 20, no. 9 (2021): e13459. 34431594 10.1111/acel.13459 PMC8441299

[23] J. Blodgett , O. Theou , S. Kirkland , P. Andreou , and K. Rockwood , “The Association Between Sedentary Behaviour, Moderate‐Vigorous Physical Activity and Frailty in NHANES Cohorts,” Maturitas 80, no. 2 (2015): 187–191. 25542406 10.1016/j.maturitas.2014.11.010

[24] J. Gao , Y. Wang , J. Xu , J. Jiang , S. Yang , and Q. Xiao , “Life Expectancy Among Older Adults With or Without Frailty in China: Multistate Modelling of a National Longitudinal Cohort Study,” BMC Medicine 21, no. 1 (2023): 101. 36927351 10.1186/s12916-023-02825-7 PMC10021933

[25] S. H. Zeisel and K. A. da Costa , “Choline: An Essential Nutrient for Public Health,” Nutrition Reviews 67, no. 11 (2009): 615–623. 19906248 10.1111/j.1753-4887.2009.00246.x PMC2782876

[26] R. Zhou , M. Yang , C. Yue , et al., “Association Between Dietary Choline Intake and Cardiovascular Diseases: National Health and Nutrition Examination Survey 2011–2016,” Nutrients 15, no. 18 (2023): 4036. 37764819 10.3390/nu15184036 PMC10534328

[27] Y. W. Zhang , P. P. Lu , Y. J. Li , et al., “Low Dietary Choline Intake Is Associated With the Risk of Osteoporosis in Elderly Individuals: A Population‐Based Study,” Food & Function 12, no. 14 (2021): 6442–6451. 34076003 10.1039/d1fo00825k

[28] C. W. Lee , E. Galvan , T. V. Lee , et al., “Low Intake of Choline Is Associated With Diminished Strength and Lean Mass Gains in Older Adults,” Journal of Frailty & Aging 12, no. 1 (2023): 78–83. 36629089 10.14283/jfa.2022.50

[29] C. W. Lee , T. V. Lee , E. Galvan , et al., “The Effect of Choline and Resistance Training on Strength and Lean Mass in Older Adults,” Nutrients 15, no. 18 (2023): 3874. 37764658 10.3390/nu15183874 PMC10534351

[30] P. Mone , V. Trimarco , U. Kansakar , R. Izzo , G. Santulli , and B. Trimarco , “Combining Choline Bitartrate and Vitamin B12 Ameliorates Cognitive Impairment in Hypertensive Elders With Cognitive Frailty,” Pharmacological Research 201 (2024): 107103. 38336310 10.1016/j.phrs.2024.107103 PMC11380760

[31] T. Tanaka , S. A. Talegawkar , Y. Jin , et al., “Metabolomic Profile of Different Dietary Patterns and Their Association With Frailty Index in Community‐Dwelling Older Men and Women,” Nutrients 14, no. 11 (2022): 2237. 35684039 10.3390/nu14112237 PMC9182888

[32] N. Rashidi Pour Fard , F. Amirabdollahian , and F. Haghighatdoost , “Dietary Patterns and Frailty: A Systematic Review and Meta‐Analysis,” Nutrition Reviews 77, no. 7 (2019): 498–513. 31038679 10.1093/nutrit/nuz007

[33] T. Tanaka , J. K. Das , Y. Jin , et al., “Plant Protein but Not Animal Protein Consumption Is Associated With Frailty Through Plasma Metabolites,” Nutrients 15, no. 19 (2023): 4193. 37836476 10.3390/nu15194193 PMC10574762

[34] M. Sotos‐Prieto , E. A. Struijk , T. T. Fung , et al., “Association Between the Quality of Plant‐Based Diets and Risk of Frailty,” Journal of Cachexia, Sarcopenia and Muscle 13, no. 6 (2022): 2854–2862. 36177985 10.1002/jcsm.13077 PMC9745455

[35] Y. Yuan , S. Chen , C. Lin , et al., “Association of Triglyceride‐Glucose Index Trajectory and Frailty in Urban Older Residents: Evidence From the 10‐Year Follow‐Up in a Cohort Study,” Cardiovascular Diabetology 22, no. 1 (2023): 264. 37775740 10.1186/s12933-023-02002-3 PMC10542691

[36] I. H. Ulus and R. J. Wurtman , “Choline Increases Acetylcholine Release,” Lancet 1, no. 8533 (1987): 624. 10.1016/s0140-6736(87)90258-3 2881154

[37] A. Moretti , M. Paoletta , S. Liguori , M. Bertone , G. Toro , and G. Iolascon , “Choline: An Essential Nutrient for Skeletal Muscle,” Nutrients 12, no. 7 (2020): 2144. 32708497 10.3390/nu12072144 PMC7400816

[38] K. D. Corbin and S. H. Zeisel , “Choline Metabolism Provides Novel Insights Into Nonalcoholic Fatty Liver Disease and Its Progression,” Current Opinion in Gastroenterology 28, no. 2 (2012): 159–165. 22134222 10.1097/MOG.0b013e32834e7b4b PMC3601486

[39] Y. Qiu , S. Liu , L. Hou , et al., “Supplemental Choline Modulates Growth Performance and Gut Inflammation by Altering the Gut Microbiota and Lipid Metabolism in Weaned Piglets,” Journal of Nutrition 151, no. 1 (2021): 20–29. 33245135 10.1093/jn/nxaa331

[40] Z. S. Choline , “Other Methyl‐Donors and Epigenetics,” Nutrients 9, no. 5 (2017): 445. 28468239 10.3390/nu9050445 PMC5452175

