# Supplementary material for: Association of Dietary Choline Intake With Incidence of Frailty: A Nationwide Prospective Cohort Study From China
Source: J Cachexia Sarcopenia Muscle. 2025 Apr 6;16(2):e13796. doi: 10.1002/jcsm.13796 (PMC11972690; doi:10.1002/jcsm.13796)
Supplement: Supplementary file 1 — Figure S1. Flowchart of recruitment and follow‐up. Figure S2. Dose‐respond associations of choline‐contributing compounds and betaine intake with incident frailty. Figure S3. Distribution of total choline intake from various food sources. Table S1. The 27 items and cut‐points for the frailty index. Table S2. Baseline characteristics of participants by quartiles of total choline intake. Table S3. Associations of lipid‐ and water‐soluble choline intake with incident pre‐frailty and frailty. Table S4. Associations of choline‐contributing compounds and betaine intake with incident pre‐frailty. Table S5. Associations of choline‐contributing compounds and betaine intake with incident (pre‐) frailty. Table S6. Associations of choline intake from different food sources with incident pre‐frailty and frailty. Table S7. Associations of total choline intake with incident frailty: subgroup analysis stratified by sex, age and energy intake. Table S8. Associations of total choline with incident pre‐frailty: subgroup analysis stratified by sex, age and energy intake. Table S9. Associations of total choline intake with incident (pre‐) frailty: subgroup analysis stratified by sex, age and energy intake. Table S10. Associations of total choline intake with incident frailty: sensitivity analysis. Table S11. Characteristics of participants for lipid profiles. [file JCSM-16-e13796-s001.docx]

**Supplement Contents**

**Supplement Methods 1**

**Supplement Methods 2**

**Supplement Methods 3**

**Figure S1.** Flowchart of recruitment and follow-up

**Figure S2.** Dose-respond associations of choline-contributing compounds and betaine intake with incident frailty

**Figure S3.** Distribution of total choline intake from various food sources

**Table S1.** The 27 items and cut-points for the frailty index

**Table S2.** Baseline characteristics of participants by quartiles of total choline intake

**Table S3.** Associations of lipid- and water-soluble choline intake with incident pre-frailty and frailty

**Table S4.** Associations of choline-contributing compounds and betaine intake with incident pre-frailty

**Table S5.** Associations of choline-contributing compounds and betaine intake with incident (pre-) frailty

**Table S6.** Associations of choline intake from different food sources with incident pre-frailty and frailty

**Table S7.** Associations of total choline intake with incident frailty: subgroup analysis stratified by sex, age and energy intake

**Table S8.** Associations of total choline with incident pre-frailty: subgroup analysis stratified by sex, age and energy intake

**Table S9.** Associations of total choline intake with incident (pre-) frailty: subgroup analysis stratified by sex, age and energy intake

**Table S10.** Associations of total choline intake with incident frailty: sensitivity analysis

**Table S11.** Characteristics of participants for lipid profiles

**Supplement Reference**

**Supplement Methods 1**

The construction of the frailty index (FI) comprises 27 items across 6 dimensions: history of chronic diseases (based on self-reported physician diagnoses), symptoms experienced in the last four weeks (based on self-reported physician diagnoses), anthropometry indexes (measured by trained technicians using standard methods), health status (self-reported), physical activity level (self-reported) and sleep duration (self-reported). The anthropometry indexes include blood pressure (BP), body weight, height, waist circumference (WC), and hip circumference, all measured by trained technicians using standardized procedure. BP was assessed three times with a standard mercury sphygmomanometer. We used the mean of measurements of systolic blood pressure (SBP) and diastolic blood pressure (DBP) in our study. Body weight was measured in light clothing (to the nearest 0.1 kg) and height barefoot (to the nearest 0.1 cm). Body mass index (BMI) was calculated as weight (kg) divided by square of height (m^2^). WC was assessed at the midpoint between the lower ribs and the iliac crest, while hip circumference was measured at the level of the greater trochanter. The waist-to-hip ratio (WHR) was calculated as the ratio of waist circumference to hip circumference, both measured in centimeters. Physical activity includes four categories: domestic, occupational, transportation and leisure types, and assessed in metabolic equivalent (MET) hours per week to consider the intensity and duration of activities [S13]. The physical activity level was calculated by multiplying the duration of each activity by the corresponding MET values based on previous study [S14].

**Supplement Methods 2**

The covariate information for this study was collected through structured questionnaires and physical measurements. The details of determining on each covariate were as follow: Age was derived from the difference between the interview dates and verified birth dates. Sex was categorized as “males” and “females” according to the sex of the interviewee at the survey. Nationality was categorized as “Han” and “minority” based on the ethnic background of the interviewee at the survey. Residence was classified as “rural” and “urban” based on the living situation of interviewee at the survey. Geographical region was classified as “northeastern China”, “eastern China”, “central China”, “southern China”, and “southwestern China” based on the province or municipality where the interviewee resided at the survey. Marital status was categorized as “married”, “never married”, and “divorced, widowed or separated” by the question “What is your current marital status?”. Education was categorized as “at or below primary school”, “middle school”, and “at or above high school” by the question “What is the highest level of education?”. Household per capita annual income is calculated by dividing the total income of surveyed households by the number of household members. Medical insurance was categorized as “yes” and “no” by the question “Whether you have medical insurance?”. Drinking was assessed through the question, “Over the past year, what was your frequency and quantity of consumption of beer, wine, and liquor?”. The alcohol concentration was consistent with previous study: beer = 4%, wine = 10%, and liquor = 38% (1 bottle = 600 mL, 1 Liang = 50 mL) [S4]. Following WHO guidelines, low-risk alcohol consumption was defined as < 21 g/d for females and < 41 g/d for males. Drinking status was categorized as: never (0 g/day); moderate (1-20 g/d for females, 1-40 g/d for males); and excess (≥ 21 g/d for females, ≥ 41 g/d for males) [S5]. Smoking was categorized as: never, previous, and current. “Never” was defined as a negative response to the question“Have you ever smoked a cigarette?”. “Current” was defined as an affirmative response to the question “Do you still smoke?”. “Previous” referred to participants who had smoked in the past but were not currently smoking. Drinking water sources were classified based on the question,“What is the source of your drinking water?”. Tap water included indoor and in-yard tap water; well water referred to in-yard well water; and other sources were categorized as other. Cooking fuel type was assessed using the question, “What type of fuel does your household typically use for cooking?”. Clean fuel included electricity, liquified petroleum gas, and natural gas, while polluting fuel included coal, charcoal, kerosene, wood, straws, and sticks [S6]. Sedentary behavior was evaluated through the question, “Do you engage in this sedentary activity?”. Participants who with a positive answer were considered to have sedentary behavior. Subsequently, participants were queried about the time of sedentary behavior during the week, “How much time do you spend from Monday to Friday and from Saturday to Sunday?”. Sedentary behavior was divided into four types: (1) watching television or videos; (2) computer usage; (3) gaming; and (4) reading. The workday sedentary behavior was derived by multiplying the daily average time spent in sedentary behavior from Monday to Friday by five. The weekend sedentary behavior was calculated as twice the daily average time spent in sedentary behavior over Saturday and Sunday. The total sedentary behavior was calculated by summing the workday and weekend sedentary behavior duration [S15]. Body weight and height were measured by trained technicians utilizing standardized methods. Body weight was measured in light clothing (to the nearest 0.1 kg) and height barefoot (to the nearest 0.1 cm). BMI was calculated by dividing weight (kg) by height squared (m^2^).

Hypertension was defined according to the following criteria: (1) mean SBP ≥ 140 mmHg and/or mean DBP ≥ 90 mmHg; (2) self-reported diagnosis of hypertension; (3) use of antihypertensive medication. Trained physicians utilized a standard mercury sphygmomanometer to measure BP in accordance with established protocols. We used the mean of three of three measurements of SBP and DBP in our study. Dietary intake assessment was conducted using a combination of detailed food consumption data collected through consecutive three 24-hour dietary recalls (24h-DRs) at the individual level, combined with a household-level food inventory weighting method over the same three-day period for each survey round [S16]. Nutrient intake was estimated by multiplying the quantity of each food item consumed by the nutrient content specified for a standard portion size of 100 g, as referenced in the Chinese Food Composition Tables (CFCT) [S7, S8]. Scores for health infrastructure, sanitation and social services were used to assess urban features in China, based on an established urbanization scale [S17]. These scores were derived from established algorithms, with a maximum of 10 points allocated. Health infrastructure was defined by the quantity and types of health facilities within 12 kilometers of the community, as well as the number of pharmacies. Sanitation was measured by the percentage of households with access to treated water and those without visible excreta outside. Social services included the availability of preschool for children under 3 years old and community-offered commercial or free medical insurance, including for women and children [S1].

**Supplement Methods 3**

Considering that lipid profiles were obtained exclusively during the 2009 survey wave, we conducted several sensitivity analyses to evaluate the impact of lipid levels on frailty. First, we employed the Multiple Imputation by Chained Equations (MICE) method to generate 10 datasets, incorporating all variables, including the outcome variables, to ensure comprehensive imputation of the missing lipid profiles. Second, we analysed only those participants with available lipid profile data (n=5876). Additionally, we conducted analyses with lipid profiles and blood pressure as both categorical and continuous covariates. All blood samples were analyzed at a nationally accredited central laboratory in Beijing, which holds ISO 15189:2007 certification for medical laboratories and strictly adheres to quality control protocols. All lipid profiles measurements were performed using the Hitachi 7600 automated analyzer (Hitachi, Inc., Tokyo, Japan) [S18]. Hyperlipidemia is defined as the presence of one or more of the following criteria in the patient: (1) total cholesterol ≥ 6.22 mmol/L; (2) triglycerides ≥ 2.26 mmol/L; (3) high-density lipoprotein cholesterol < 1.04 mmol/L; (4) low-density lipoprotein cholesterol ≥ 4.14 mmol/L [S19].


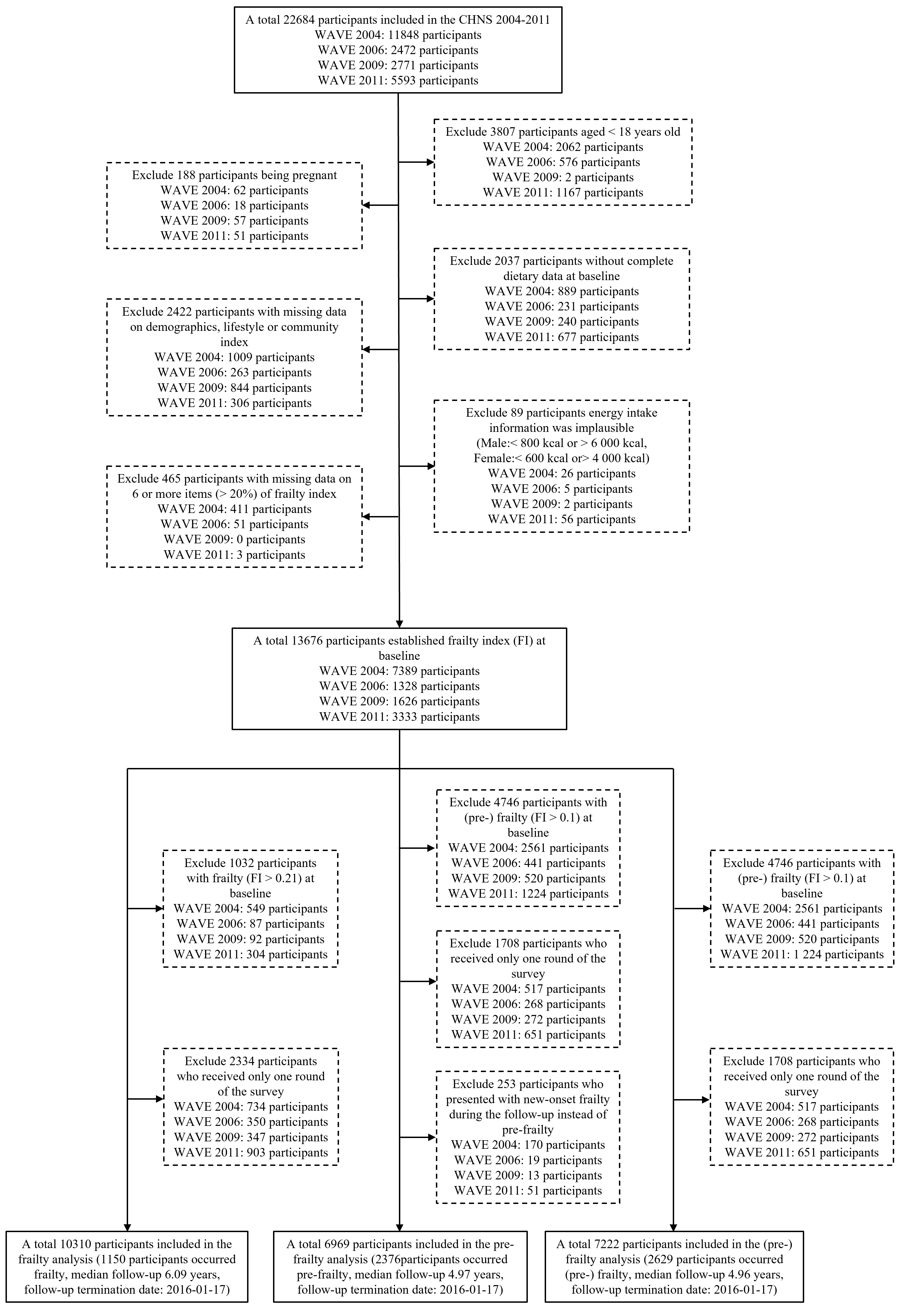


**Figure S1. Flowchart of recruitment and follow-up**

* The term (pre-) frailty refers to the combined states of pre-frailty and frailty.


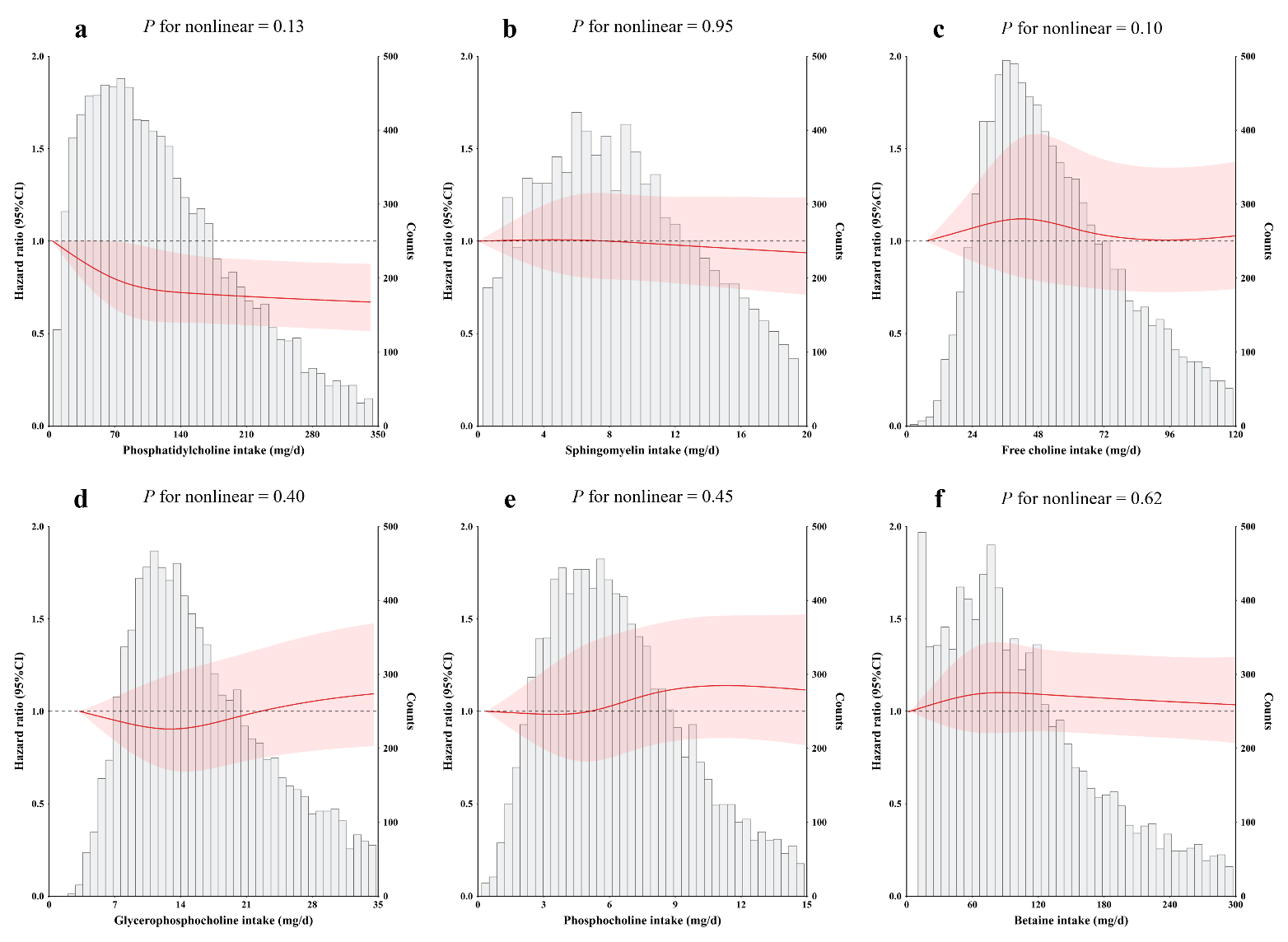


**Figure S2. Dose-respond associations of choline-contributing compounds and betaine intake with incident frailty**

Adjusted for age (years, continuous), sex (female or male), nationality (Han or minority), residence (urban or rural), geographical region (northeastern China, eastern China, central China, southern China or southwestern China), marital status (married, single, or divorced, widowed or separated), education (at or below primary school, middle school, or at or above high school), household per capita annual income (CNY, continuous), medical insurance (yes or no), drinking (never, moderate, or excess), smoking (never, previous, or current), drinking water source (tap water, well water or other), cooking fuel type (clean fuel or polluting fuel), sedentary behavior (hours/d, continuous), dietary intake of energy (kcal/d, continuous), protein (g/d, continuous), fat (g/d, continuous), and carbohydrate (g/d, continuous), body mass index (kg/m^2^, continuous), hypertension (yes or no), health infrastructure score (continuous), sanitation score (continuous), and social services score (continuous).


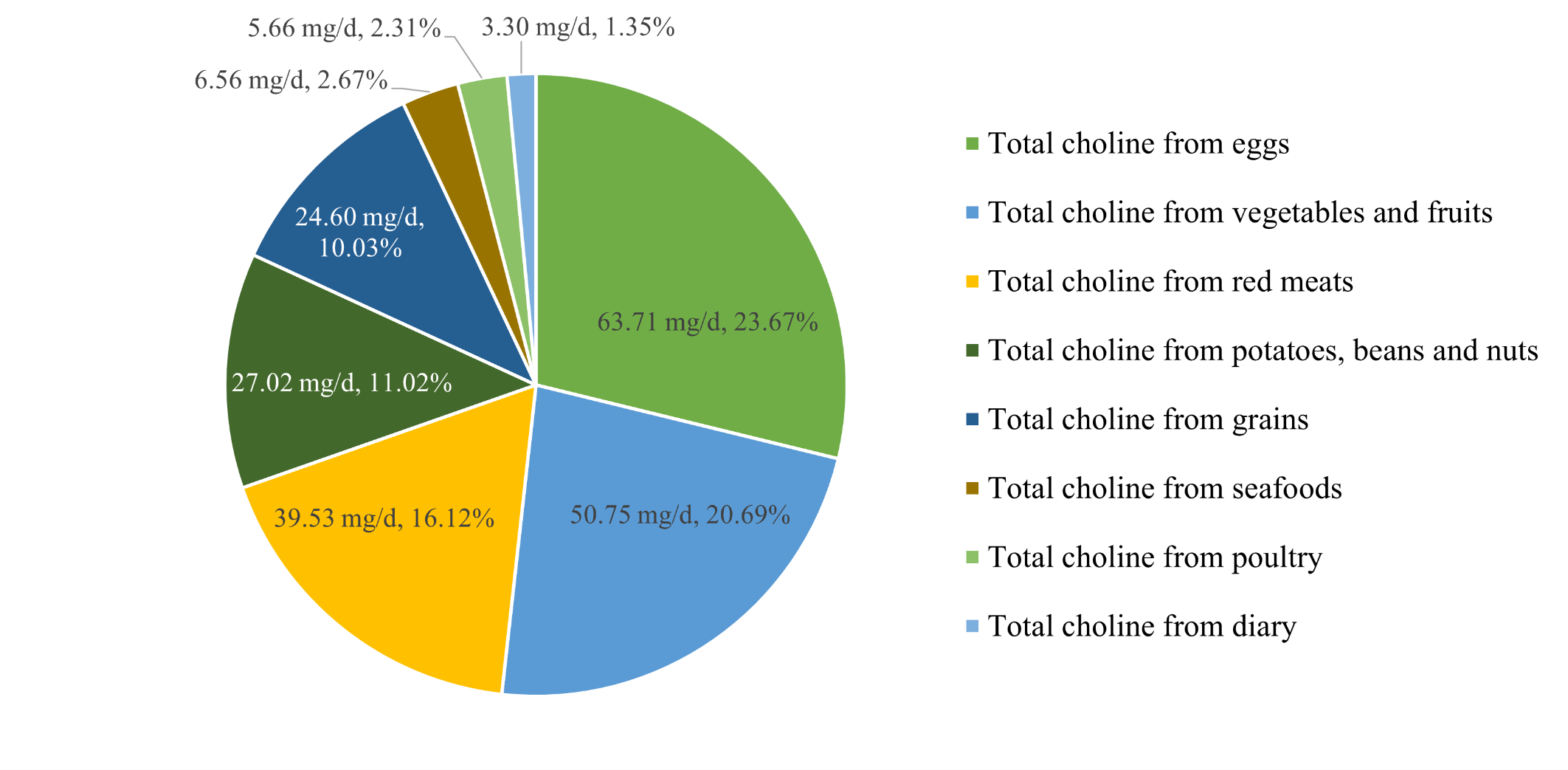


**Figure S3. Distribution of total choline intake from various food sources**

**Note:** The values represent the mean choline intake (mg/d) from each food source, with percentages indicating the proportion of total choline intake derived from each respective source.

| **Table S1. The 27 items and cut-points for the frailty index** | |
| --- | --- |
| **Items included in the frailty index** | **Cut-points** |
| **History of chronic disease** |  |
| 1. Self-reported physician diagnosed hypertension /  Self-reported take anti- hypertension drugs | Yes = 1, No = 0 |
| 2. Self-reported physician diagnosed diabetes | Yes = 1, No = 0 |
| 3. Self-reported physician diagnosed myocardial infarct | Yes = 1, No = 0 |
| 4. Self-reported physician diagnosed stroke | Yes = 1, No = 0 |
| 5. Self-reported physician diagnosed bone fracture | Yes = 1, No = 0 |
| 6. Self-reported physician diagnosed asthma | Yes = 1, No = 0 |
| 7. Self-reported physician diagnosed cancer | Yes = 1, No = 0 |
| 8. Self-reported physician diagnosed monocular / bilateral blindness | Yes = 1, No = 0 |
| 9. Self-reported physician diagnosed unilateral / bilateral limb amputation or unilateral / bilateral loss of limb function | Yes = 1, No = 0 |
| 10. Self-reported physician diagnosed unilateral / bilateral leg amputation or unilateral / bilateral loss of leg function | Yes = 1, No = 0 |
| **Symptoms experienced in the last 4 weeks** |  |
| 1. Self-reported physician diagnosed fever, sore throat, or cough | Yes = 1, No = 0 |
| 2. Self-reported physician diagnosed diarrhea or stomachache | Yes = 1, No = 0 |
| 3. Self-reported physician diagnosed headache or dizziness | Yes = 1, No = 0 |
| 4. Self-reported physician diagnosed joint or muscle aches | Yes = 1, No = 0 |
| 5. Self-reported physician diagnosed rash or skin dermatitis | Yes = 1, No = 0 |
| 6. Self-reported physician diagnosed eye or ear diseases | Yes = 1, No = 0 |
| 7. Self-reported physician diagnosed heart disease or chest pain | Yes = 1, No = 0 |
| 8. Self-reported physician diagnosed other infections or diseases | Yes = 1, No = 0 |
| 9. Self-reported physician diagnosed other chronic diseases | Yes = 1, No = 0 |
| **Anthropometry index** |  |
| 1. Systolic blood pressure (SBP, mmHg) | SBP < 90 = 1, SBP ≥ 140 = 1, 90 ≤ SBP < 140 = 0 |
| 2. Diastolic blood pressure (DBP, mmHg) | DBP < 60 = 1, DBP ≥ 90 = 1, 60 ≤ DBP < 90 = 0 |
| 3. Body mass index (BMI, kg/m^2^) | BMI < 18.5 = 1, BMI ≥ 28 = 1, 24 ≤ BMI < 28 = 0.5, 18.5 ≤ BMI < 24 = 0 |
| 4. Waist-to-hip ratio (WHR) | Female: WHR ≥ 0.90 = 1, 0.85 ≤ WHR < 0.90 = 0.5, WHR < 0.85 = 0  Male: WHR ≥ 0.95 = 1, 0.90 ≤ WHR < 0.95 = 0.5, WHR < 0.90 = 0 |
| **Health status** |  |
| 1. Self-reported maintained the same level of energy as last year | Disagree = 1, Neutral = 0.5, Agree = 0 |
| 2. Self-reported current health condition | Poor = 1, Fair = 0.5, Good = 0, Excellent = 0 |
| **Physical activity level** | Low level = 1, Moderate level = 0.5, High level = 0 |
| **Sleep duration (hours/d)** | Less than 7 =1, More than 8 =1, 7~8 = 0 |

| **Table S2. Baseline characteristics of participants by quartiles of total choline intake** | | | | | |
| --- | --- | --- | --- | --- | --- |
| **Characteristic** | **Total** | **Total choline intake quartiles** | | | |
|  |  | **1** | **2** | **3** | **4** |
| **Participants, No. (%)** | 7222 (100.0) | 1806 (25.0) | 1805 (25.0) | 1805 (25.0) | 1806 (25.0) |
| **Age (years), mean (SD)** | 43.3 (13.5) | 44.2 (14.3) | 43.1 (13.1) | 43.4 (13.2) | 42.4 (13.1) |
| 18-44, No. (%) | 4035 (55.9) | 963 (53.3) | 1024 (56.7) | 1010 (56.0) | 1038 (57.5) |
| 45-59, No. (%) | 2317 (32.1) | 574 (31.8) | 576 (31.9) | 582 (32.2) | 585 (32.4) |
| ≥ 60, No. (%) | 870 (12.0) | 269 (14.9) | 205 (11.4) | 213 (11.8) | 183 (10.1) |
| **Female, No. (%)** | 3750 (51.9) | 1019 (56.4) | 992 (55.0) | 935 (51.8) | 804 (44.5) |
| **Nationality, No. (%)** |  |  |  |  |  |
| Han | 6345 (87.9) | 1525 (84.4) | 1580 (87.5) | 1614 (89.4) | 1626 (90.0) |
| Minority | 877 (12.1) | 281 (15.6) | 225 (12.5) | 191 (10.6) | 180 (10.0) |
| **Residence, No. (%)** |  |  |  |  |  |
| Rural | 4731 (65.5) | 1410 (78.1) | 1284 (71.1) | 1137 (63.0) | 900 (49.8) |
| Urban | 2491 (34.5) | 396 (21.9) | 521 (28.9) | 668 (37.0) | 906 (50.2) |
| **Geographical region, No. (%)** |  |  |  |  |  |
| Northeastern China | 1637 (22.7) | 259 (14.3) | 370 (20.5) | 438 (24.3) | 570 (31.6) |
| Eastern China | 1772 (24.5) | 314 (17.4) | 400 (22.2) | 508 (28.1) | 550 (30.5) |
| Central China | 1157 (16.0) | 322 (17.8) | 323 (17.9) | 276 (15.3) | 236 (13.1) |
| Southern China | 1514 (21.0) | 502 (27.8) | 421 (23.3) | 332 (18.4) | 259 (14.3) |
| Southwestern China | 1142 (15.8) | 409 (22.6) | 291 (16.1) | 251 (13.9) | 191 (10.6) |
| **Marital status, No. (%)** |  |  |  |  |  |
| Married | 6234 (86.3) | 1548 (85.7) | 1550 (85.9) | 1573 (87.1) | 1563 (86.5) |
| Never married | 635 (8.8) | 143 (7.9) | 153 (8.5) | 159 (8.8) | 180 (10.0) |
| Divorced, widowed or separated | 353 (4.9) | 115 (6.4) | 102 (5.7) | 73 (4.0) | 63 (3.5) |
| **Education, No. (%)** |  |  |  |  |  |
| At or below primary school | 2395 (33.2) | 863 (47.8) | 640 (35.5) | 512 (28.4) | 380 (21.0) |
| Middle school | 3641 (50.4) | 825 (45.7) | 924 (51.2) | 938 (52.0) | 954 (52.8) |
| At or above high school | 1186 (16.4) | 118 (6.5) | 241 (13.4) | 355 (19.7) | 472 (26.1) |
| **Household per capita annual income (CNY), mean (SD)** | 8830.9 (12601.2) | 5353.4  (9971.9) | 7785.3  (10788.7) | 10103.1 (13726.4) | 12081.8 (14345.0) |
| **Medical insurance, No. (%)** | 3448 (47.7) | 600 (33.2) | 831 (46.0) | 950 (52.6) | 1067 (59.1) |
| **Drinking, No. (%)** |  |  |  |  |  |
| Never | 5137 (71.1) | 1414 (78.3) | 1305 (72.3) | 1253 (69.4) | 1165 (64.5) |
| Moderate | 1616 (22.4) | 310 (17.2) | 399 (22.1) | 425 (23.5) | 482 (26.7) |
| Excess | 1616 (22.4) | 310 (17.2) | 399 (22.1) | 425 (23.5) | 482 (26.7) |
| **Smoking, No. (%)** |  |  |  |  |  |
| Never | 4911 (68.0) | 1271 (70.4) | 1266 (70.1) | 1220 (67.6) | 1154 (63.9) |
| Previous | 192 (2.7) | 43 (2.4) | 41 (2.3) | 51 (2.8) | 57 (3.2) |
| Current | 2119 (29.3) | 492 (27.2) | 498 (27.6) | 534 (29.6) | 595 (32.9) |
| **Drinking water source, No. (%)** |  |  |  |  |  |
| Tap water | 5699 (78.9) | 1225 (67.8) | 1429 (79.2) | 1478 (81.9) | 1567 (86.8) |
| Well water | 1319 (18.3) | 477 (26.4) | 334 (18.5) | 295 (16.3) | 213 (11.8) |
| Other | 204 (2.8) | 104 (5.8) | 42 (2.3) | 32 (1.8) | 26 (1.4) |
| **Cooking fuel type, No. (%)** |  |  |  |  |  |
| Clean fuel | 3988 (55.2) | 679 (37.6) | 933 (51.7) | 1095 (60.7) | 1281 (70.9) |
| Polluting fuel | 3234 (44.8) | 1127 (62.4) | 872 (48.3) | 710 (39.3) | 525 (29.1) |
| **Sedentary behavior (hours/d), mean (SD)** | 1.7 (1.5) | 1.7 (1.5) | 1.8 (1.5) | 1.8 (1.5) | 1.8 (1.5) |
| **BMI (****kg/m^2^), mean (SD)** | 22.7 (3.0) | 22.4 (3.0) | 22.7 (3.0) | 22.9 (3.0) | 23.0 (3.1) |
| **SBP (mmHg), mean (SD)** | 117.1 (12.6) | 117.0 (13.1) | 116.7 (12.7) | 117.2 (12.3) | 117.6 (12.2) |
| **DBP (mmHg), mean (SD)** | 76.3 (8.4) | 76.0 (8.8) | 76.1 (8.5) | 76.6 (8.2) | 76.7 (8.1) |
| **Hypertension, No. (%)** | 3387 (46.9 | 831 (46.0) | 824 (45.7) | 861 (47.7) | 871 (48.2) |
| **Dietary intake** |  |  |  |  |  |
| Energy (kcal/d), mean (SD) | 2114.5 (659.8) | 1888.9 (585.5) | 2028.2 (621.9) | 2157.5 (649.4) | 2383.2 (676.9) |
| Protein (g/d), mean (SD) | 66.7 (24.3) | 51.1 (15.5) | 61.6 (19.2) | 70.0 (22.3) | 84.2 (25.9) |
| Fat (g/d), mean (SD) | 70.6 (37.4) | 53.5 (30.7) | 65.6 (34.1) | 74.8 (35.1) | 88.6 (39.8) |
| Carbohydrate (g/d), mean (SD) | 299.9 (115.8) | 299.7 (110.5) | 295.8 (119.7) | 297.7 (119.4) | 306.5 (113.2) |
| **Community index** |  |  |  |  |  |
| Health infrastructure score, mean (SD) | 5.4 (2.4) | 4.9 (2.3) | 5.5 (2.3) | 5.5 (2.4) | 5.9 (2.4) |
| Sanitation score, mean (SD) | 6.8 (2.9) | 5.8 (3.0) | 6.8 (2.9) | 7.2 (2.8) | 7.7 (2.6) |
| Social services score, mean (SD) | 3.5 (3.0) | 2.8 (2.7) | 3.5 (3.0) | 3.8 (3.1) | 4.1 (3.2) |
| **Total choline (mg/d), mean (SD)** | 244.7 (184.6) | 98.1 (26.9) | 171.9 (19.9) | 249.2 (26.0) | 459.4 (248.2) |
| Lipid-soluble choline^1^ (mg/d), mean (SD) | 153.1 (140.3) | 46.4 (21.9) | 99.1 (27.3) | 156.1 (38.0) | 310.9 (192.1) |
| Water-soluble choline^2^ (mg/d), mean (SD) | 91.8 (58.6) | 53.5 (16.8) | 74.4 (23.7) | 94.4 (32.4) | 144.8 (85.1) |
| Phosphatidylcholine (mg/d), mean (SD) | 143.2 (135.5) | 42.3 (20.0) | 91.5 (25.3) | 145.2 (35.8) | 294.0 (188.3) |
| Sphingomyelin (mg/d), mean (SD) | 9.9 (7.5) | 4.2 (3.4) | 7.6 (4.4) | 10.9 (5.4) | 16.9 (8.9) |
| Free choline (mg/d), mean (SD) | 64.4 (46.3) | 37.1 (14.3) | 52.4 (21.6) | 66.4 (29.4) | 101.7 (69.0) |
| Glycerophosphocholine (mg/d), mean (SD) | 20.4 (16.2) | 11.4 (4.6) | 15.7 (6.3 | 20.5 (8.9) | 33.9 (25.1) |
| Phosphocholine (mg/d), mean (SD) | 7.0 (4.2) | 5.0 (2.9) | 6.2 (3.3) | 7.5 (3.9) | 9.2 (5.3) |
| **Betaine (mg/d), mean (SD)** | 116.8 (107.6) | 108.6 (109.8) | 113.6 (109.5) | 117.5 (101.5) | 127.4 (108.8) |

^1^ Lipid-soluble choline includes phosphatidylcholine and sphingomyelin.

^2^ Water-soluble choline includes free choline, glycerophosphocholine and phosphocholine.

The mean of three measurements of systolic and diastolic blood pressure was used.

BMI: body mass index, SBP: systolic blood pressure, and DBP: diastolic blood pressure.

**Table S3. Associations of** **lipid- and water-soluble choline intake with incident pre-frailty and frailty**

| **Outcomes** | **Lipid- and water-soluble choline intake quartiles** | | | |
| --- | --- | --- | --- | --- |
|  | **1** | **2** | **3** | **4** |
| **Frailty (n=10310)** |  |  |  |  |
| **Lipid-soluble choline^1^** |  |  |  |  |
| Mean intake (SD), mg/d | 41.67 (17.05) | 95.09 (15.46) | 156.32 (20.95) | 322.34 (255.25) |
| Events/participants | 368/2578 | 274/2577 | 256/2578 | 252/2577 |
| Model 1^a^, HR (95% CI) | 1 (reference) | 0.84 (0.72, 0.98) | 0.83 (0.71, 0.97) | 0.85 (0.72, 1.00) |
| Model 2^b^, HR (95% CI) | 1 (reference) | 0.81 (0.69, 0.95) | 0.82 (0.69, 0.98) | 0.77 (0.63, 0.95) |
| Model 3^c^, HR (95% CI) | 1 (reference) | 0.80 (0.68, 0.95) | 0.82 (0.69, 0.98) | 0.77 (0.63, 0.95) |
| **Water-soluble choline^2^** |  |  |  |  |
| Mean intake (SD), mg/d | 43.48 (9.47) | 66.80 (6.33) | 92.45 (9.01) | 163.47 (83.88) |
| Events/participants | 309/2578 | 297/2577 | 276/2577 | 268/2578 |
| Model 1^a^, HR (95% CI) | 1 (reference) | 0.95 (0.81, 1.11) | 0.89 (0.76, 1.05) | 0.81 (0.69, 0.95) |
| Model 2^b^, HR (95% CI) | 1 (reference) | 1.02 (0.86, 1.20) | 1.04 (0.87, 1.24) | 1.02 (0.83, 1.24) |
| Model 3^c^, HR (95% CI) | 1 (reference) | 1.01 (0.85, 1.19) | 1.04 (0.87, 1.25) | 1.02 (0.84, 1.25) |
| **Pre-frailty (n=6969)** |  |  |  |  |
| **Lipid-soluble choline^1^** |  |  |  |  |
| Mean intake (SD), mg/d | 42.89 (17.20) | 96.10 (15.12) | 156.44 (20.61) | 319.13 (189.14) |
| Events/participants | 688/1743 | 588/1742 | 544/1742 | 556/1742 |
| Model 1^a^, HR (95% CI) | 1 (reference) | 0.93 (0.83, 1.03) | 0.88 (0.79, 0.99) | 0.95 (0.85, 1.06) |
| Model 2^b^, HR (95% CI) | 1 (reference) | 0.95 (0.85, 1.07) | 0.89 (0.79, 1.00) | 0.97 (0.85, 1.12) |
| Model 3^c^, HR (95% CI) | 1 (reference) | 0.95 (0.85, 1.07) | 0.88 (0.77, 0.99) | 0.96 (0.84, 1.11) |
| **Water-soluble choline^2^** |  |  |  |  |
| Mean intake (SD), mg/d | 44.52 (9.33) | 67.81 (6.45) | 93.50 (9.01) | 162.75 (76.16) |
| Events/participants | 634/1743 | 557/1742 | 578/1742 | 607/1742 |
| Model 1^a^, HR (95% CI) | 1 (reference) | 0.83 (0.74, 0.94) | 0.88 (0.79, 0.99) | 0.88 (0.79, 0.99) |
| Model 2^b^, HR (95% CI) | 1 (reference) | 0.82 (0.73, 0.92) | 0.90 (0.80, 1.02) | 0.93 (0.81, 1.06) |
| Model 3^c^, HR (95% CI) | 1 (reference) | 0.83 (0.74, 0.94) | 0.91 (0.81, 1.03) | 0.95 (0.83, 1.08) |
| **(Pre-) frailty* (n=7222)** |  |  |  |  |
| **Lipid-soluble choline^1^** |  |  |  |  |
| Mean intake (SD), mg/d | 42.56 (17.05) | 95.56 (15.22) | 156.12 (20.64) | 318.25 (187.13) |
| Events/participants | 764/1806 | 647/1805 | 602/1805 | 616/1806 |
| Model 1^a^, HR (95% CI) | 1 (reference) | 0.91 (0.82, 1.01) | 0.87 (0.78, 0.97) | 0.94 (0.84, 1.04) |
| Model 2^b^, HR (95% CI) | 1 (reference) | 0.94 (0.84, 1.05) | 0.88 (0.78, 0.99) | 0.97 (0.85, 1.11) |
| Model 3^c^, HR (95% CI) | 1 (reference) | 0.93 (0.83, 1.04) | 0.87 (0.77, 0.98) | 0.96 (0.84, 1.10) |
| **Water-soluble choline^2^** |  |  |  |  |
| Mean intake (SD), mg/d | 44.31 (9.35) | 67.47 (6.40) | 93.09 (9.02) | 162.24 (75.44) |
| Events/participants | 709/1806 | 624/1805 | 636/1805 | 660/1806 |
| Model 1^a^, HR (95% CI) | 1 (reference) | 0.85 (0.76, 0.94) | 0.87 (0.78, 0.97) | 0.86 (0.77, 0.96) |
| Model 2^b^, HR (95% CI) | 1 (reference) | 0.85 (0.76, 0.95) | 0.92 (0.82, 1.03) | 0.92 (0.81, 1.05) |
| Model 3^c^, HR (95% CI) | 1 (reference) | 0.86 (0.77, 0.97) | 0.94 (0.83, 1.05) | 0.94 (0.83, 1.07) |

* The term (pre-) frailty refers to the combined states of pre-frailty and frailty.

^1^ Lipid-soluble choline includes phosphatidylcholine and sphingomyelin.

^2^ Water-soluble choline includes free choline, glycerophosphocholine and phosphocholine.

^a^ The crude model was not adjusted for any confounders.

^b^ Adjusted for age (years, continuous), sex (female or male), nationality (Han or minority), residence (urban or rural), geographical region (northeastern China, eastern China, central China, southern China or southwestern China), marital status (married, single, or divorced, widowed or separated), education (at or below primary school, middle school, or at or above high school), household per capita annual income (CNY, continuous), medical insurance (yes or no), drinking (never, moderate, or excess), smoking (never, previous, or current), drinking water source (tap water, well water or other), cooking fuel type (clean fuel or polluting fuel), sedentary behavior (hours/d, continuous), dietary intake of energy (kcal/d, continuous), protein (g/d, continuous), fat (g/d, continuous), and carbohydrate (g/d, continuous).

^c^ Additionally adjusted for body mass index (kg/m^2^, continuous), hypertension (yes or no), health infrastructure score (continuous), sanitation score (continuous), and social services score (continuous).

| **Table S4. Associations of choline-contributing compounds and betaine intake with incident** **pre-frailty** | | | | |
| --- | --- | --- | --- | --- |
| **Dietary choline and betaine** | **Choline-contributing compounds and betaine intake quartiles** | | | |
|  | **1** | **2** | **3** | **4** |
| **Phosphatidylcholine** |  |  |  |  |
| Mean intake (SD), mg/d | 39.23 (15.67) | 88.61 (14.09) | 145.30 (19.57) | 301.74 (185.81) |
| Events/participants | 691/1743 | 584/1742 | 539/1742 | 562/1742 |
| Model 1^a^, HR (95% CI) | 1 (reference) | 0.91 (0.81, 1.01) | 0.85 (0.76, 0.95) | 0.95 (0.85, 1.06) |
| Model 2^b^, HR (95% CI) | 1 (reference) | 0.93 (0.83, 1.05) | 0.85 (0.75, 0.96) | 0.96 (0.84, 1.10) |
| Model 3^c^, HR (95% CI) | 1 (reference) | 0.93 (0.83, 1.04) | 0.84 (0.74, 0.95) | 0.95 (0.83, 1.09) |
| **Sphingomyelin** |  |  |  |  |
| Mean intake (SD), mg/d | 2.05 (1.57) | 6.70 (1.15) | 10.98 (1.43) | 19.95 (6.81) |
| Events/participants | 717/1743 | 607/1742 | 530/1742 | 522/1742 |
| Model 1^a^, HR (95% CI) | 1 (reference) | 0.93 (0.84, 1.04) | 0.84 (0.75, 0.94) | 0.96 (0.85, 1.07) |
| Model 2^b^, HR (95% CI) | 1 (reference) | 0.93 (0.83, 1.04) | 0.85 (0.75, 0.97) | 1.02 (0.87, 1.19) |
| Model 3^c^, HR (95% CI) | 1 (reference) | 0.94 (0.84, 1.05) | 0.84 (0.74, 0.96) | 1.00 (0.85, 1.17) |
| **Free choline** |  |  |  |  |
| Mean intake (SD), mg/d | 28.01 (6.39) | 44.90 (4.70) | 64.65 (7.05) | 121.17 (60.29) |
| Events/participants | 593/1743 | 564/1742 | 595/1742 | 624/1742 |
| Model 1^a^, HR (95% CI) | 1 (reference) | 0.92 (0.82, 1.04) | 0.97 (0.86, 1.08) | 0.94 (0.84, 1.05) |
| Model 2^b^, HR (95% CI) | 1 (reference) | 0.93 (0.82, 1.04) | 0.98 (0.86, 1.11) | 1.02 (0.89, 1.16) |
| Model 3^c^, HR (95% CI) | 1 (reference) | 0.94 (0.83, 1.06) | 0.98 (0.87, 1.12) | 1.02 (0.89, 1.16) |
| **Glycerophosphocholine** |  |  |  |  |
| Mean intake (SD), mg/d | 8.77 (1.89) | 13.87 (1.41) | 19.85 (2.25) | 39.40 (22.62) |
| Events/participants | 667/1743 | 606/1742 | 559/1742 | 544/1742 |
| Model 1^a^, HR (95% CI) | 1 (reference) | 0.91 (0.82, 1.02) | 0.82 (0.74, 0.92) | 0.88 (0.79, 0.99) |
| Model 2^b^, HR (95% CI) | 1 (reference) | 0.97 (0.86, 1.08) | 0.92 (0.81, 1.04) | 1.01 (0.87, 1.16) |
| Model 3^c^, HR (95% CI) | 1 (reference) | 0.96 (0.86, 1.08) | 0.92 (0.81, 1.04) | 1.02 (0.89, 1.18) |
| **Phosphocholine** |  |  |  |  |
| Mean intake (SD), mg/d | 2.89 (0.88) | 5.15 (0.58) | 7.38 (0.77) | 12.53 (4.32) |
| Events/participants | 643/1743 | 608/1742 | 577/1742 | 548/1742 |
| Model 1^a^, HR (95% CI) | 1 (reference) | 1.00 (0.90, 1.12) | 0.99 (0.89, 1.11) | 0.97 (0.86, 1.09) |
| Model 2^b^, HR (95% CI) | 1 (reference) | 1.04 (0.93, 1.17) | 1.01 (0.90, 1.14) | 0.93 (0.81, 1.06) |
| Model 3^c^, HR (95% CI) | 1 (reference) | 1.04 (0.93, 1.17) | 1.03 (0.91, 1.16) | 0.94 (0.82, 1.07) |
| **Betaine** |  |  |  |  |
| Mean intake (SD), mg/d | 20.18 (12.46) | 67.17 (12.85) | 117.97 (18.31) | 262.38 (114.47) |
| Events/participants | 638/1743 | 546/1742 | 587/1742 | 605/1742 |
| Model 1^a^, HR (95% CI) | 1 (reference) | 0.89 (0.79, 0.99) | 0.99 (0.89, 1.11) | 0.98 (0.88, 1.09) |
| Model 2^b^, HR (95% CI) | 1 (reference) | 0.86 (0.76, 0.97) | 1.03 (0.91, 1.16) | 1.00 (0.88, 1.14) |
| Model 3^c^, HR (95% CI) | 1 (reference) | 0.86 (0.76, 0.97) | 1.01 (0.90, 1.14) | 0.94 (0.83, 1.07) |

^a^ The crude model was not adjusted for any confounders.

^b^ Adjusted for age (years, continuous), sex (female or male), nationality (Han or minority), residence (urban or rural), geographical region (northeastern China, eastern China, central China, southern China or southwestern China), marital status (married, single, or divorced, widowed or separated), education (at or below primary school, middle school, or at or above high school), household per capita annual income (CNY, continuous), medical insurance (yes or no), drinking (never, moderate, or excess), smoking (never, previous, or current), drinking water source (tap water, well water or other), cooking fuel type (clean fuel or polluting fuel), sedentary behavior (hours/d, continuous), dietary intake of energy (kcal/d, continuous), protein (g/d, continuous), fat (g/d, continuous), and carbohydrate (g/d, continuous).

^c^ Additionally adjusted for body mass index (kg/m^2^, continuous), hypertension (yes or no), health infrastructure score (continuous), sanitation score (continuous), and social services score (continuous).

| **Table S5. Associations of choline-contributing compounds and betaine intake with incident (pre-) frailty*** | | | | |
| --- | --- | --- | --- | --- |
| **Dietary choline and betaine** | **Choline-contributing compounds and betaine intake quartiles** | | | |
|  | **1** | **2** | **3** | **4** |
| **Phosphatidylcholine** |  |  |  |  |
| Mean intake (SD), mg/d | 38.93 (15.53) | 88.10 (14.17) | 145.03 (19.61) | 300.89 (183.78) |
| Events/participants | 770/1806 | 638/1805 | 598/1805 | 623/1806 |
| Model 1^a^, HR (95% CI) | 1 (reference) | 0.88 (0.79, 0.98) | 0.84 (0.76, 0.94) | 0.93 (0.84, 1.04) |
| Model 2^b^, HR (95% CI) | 1 (reference) | 0.92 (0.83, 1.03) | 0.85 (0.76, 0.96) | 0.96 (0.84, 1.09) |
| Model 3^c^, HR (95% CI) | 1 (reference) | 0.92 (0.83, 1.03) | 0.84 (0.75, 0.95) | 0.95 (0.84, 1.08) |
| **Sphingomyelin** |  |  |  |  |
| Mean intake (SD), mg/d | 2.03 (1.55) | 6.66 (1.14) | 10.94 (1.43) | 19.91 (6.83) |
| Events/participants | 792/1806 | 669/1805 | 588/1805 | 580/1806 |
| Model 1^a^, HR (95% CI) | 1 (reference) | 0.92 (0.83, 1.02) | 0.84 (0.75, 0.93) | 0.95 (0.86, 1.06) |
| Model 2^b^, HR (95% CI) | 1 (reference) | 0.91 (0.81, 1.01) | 0.85 (0.75, 0.96) | 1.02 (0.87, 1.19) |
| Model 3^c^, HR (95% CI) | 1 (reference) | 0.92 (0.82, 1.03) | 0.84 (0.75, 0.96) | 1.00 (0.86, 1.17) |
| **Free choline** |  |  |  |  |
| Mean intake (SD), mg/d | 27.85 (6.39) | 44.69 (4.65) | 64.37 (7.07) | 120.72 (59.74) |
| Events/participants | 668/1806 | 633/1805 | 646/1805 | 682/1806 |
| Model 1^a^, HR (95% CI) | 1 (reference) | 0.92 (0.83, 1.03) | 0.92 (0.83, 1.03) | 0.91 (0.82, 1.01) |
| Model 2^b^, HR (95% CI) | 1 (reference) | 0.93 (0.83, 1.04) | 0.95 (0.85, 1.07) | 1.00 (0.88, 1.13) |
| Model 3^c^, HR (95% CI) | 1 (reference) | 0.94 (0.84, 1.06) | 0.97 (0.86, 1.09) | 1.00 (0.88, 1.14) |
| **Glycerophosphocholine** |  |  |  |  |
| Mean intake (SD), mg/d | 8.71 (1.89) | 13.80 (1.40) | 19.79 (2.26) | 39.30 (22.47) |
| Events/participants | 747/1806 | 667/1805 | 615/1805 | 600/1806 |
| Model 1^a^, HR (95% CI) | 1 (reference) | 0.90 (0.81, 1.00) | 0.80 (0.72, 0.89) | 0.87 (0.78, 0.96) |
| Model 2^b^, HR (95% CI) | 1 (reference) | 0.98 (0.87, 1.09) | 0.92 (0.81, 1.03) | 1.01 (0.88, 1.16) |
| Model 3^c^, HR (95% CI) | 1 (reference) | 0.98 (0.87, 1.09) | 0.92 (0.82, 1.04) | 1.03 (0.90, 1.18) |
| **Phosphocholine** |  |  |  |  |
| Mean intake (SD), mg/d | 2.86 (0.88) | 5.14 (0.58) | 7.36 (0.77) | 12.50 (4.30) |
| Events/participants | 715/1806 | 670/1805 | 640/1805 | 604/1806 |
| Model 1^a^, HR (95% CI) | 1 (reference) | 0.98 (0.89, 1.09) | 0.98 (0.88, 1.10) | 0.95 (0.86, 1.06) |
| Model 2^b^, HR (95% CI) | 1 (reference) | 1.05 (0.94, 1.17) | 1.03 (0.91, 1.15) | 0.94 (0.83, 1.06) |
| Model 3^c^, HR (95% CI) | 1 (reference) | 1.05 (0.94, 1.17) | 1.04 (0.93, 1.17) | 0.95 (0.84, 1.08) |
| **Betaine** |  |  |  |  |
| Mean intake (SD), mg/d | 20.30 (12.56) | 67.12 (12.74) | 117.83 (18.34) | 261.87 (113.36) |
| Events/participants | 699/1806 | 618/1805 | 643/1805 | 669/1806 |
| Model 1^a^, HR (95% CI) | 1 (reference) | 0.92 (0.83, 1.03) | 0.99 (0.89, 1.10) | 0.99 (0.89, 1.10) |
| Model 2^b^, HR (95% CI) | 1 (reference) | 0.89 (0.79, 1.00) | 1.03 (0.92, 1.15) | 1.04 (0.92, 1.17) |
| Model 3^c^, HR (95% CI) | 1 (reference) | 0.89 (0.79, 0.99) | 1.01 (0.91, 1.14) | 0.97 (0.86, 1.10) |

* The term (pre-) frailty refers to the combined states of pre-frailty and frailty.

^a^ The crude model was not adjusted for any confounders.

^b^ Adjusted for age (years, continuous), sex (female or male), nationality (Han or minority), residence (urban or rural), geographical region (northeastern China, eastern China, central China, southern China or southwestern China), marital status (married, single, or divorced, widowed or separated), education (at or below primary school, middle school, or at or above high school), household per capita annual income (CNY, continuous), medical insurance (yes or no), drinking (never, moderate, or excess), smoking (never, previous, or current), drinking water source (tap water, well water or other), cooking fuel type (clean fuel or polluting fuel), sedentary behavior (hours/d, continuous), dietary intake of energy (kcal/d, continuous), protein (g/d, continuous), fat (g/d, continuous), and carbohydrate (g/d, continuous).

^c^ Additionally adjusted for body mass index (kg/m^2^, continuous), hypertension (yes or no), health infrastructure score (continuous), sanitation score (continuous), and social services score (continuous).

| **Table S6. Associations** **of** **choline intake from different** **food sources with incident pre-frailty and frailty** | | | | |
| --- | --- | --- | --- | --- |
| **Outcomes** | **Intake of choline from different food sources quartiles** | | | |
|  | **1** | **2** | **3** | **4** |
| **Frailty (n=10310)** |  |  |  |  |
| **Plant-based sources** |  |  |  |  |
| Mean intake (SD), mg/d | 39.53 (10.50) | 68.45 (8.13) | 102.77 (12.53) | 205.62 (115.99) |
| Events/participants | 296/2578 | 298/2577 | 273/2577 | 283/2578 |
| Model 1^a^, HR (95% CI) | 1 (reference) | 0.93 (0.80, 1.10) | 0.81 (0.69, 0.95) | 0.76 (0.64, 0.89) |
| Model 2^b^, HR (95% CI) | 1 (reference) | 0.99 (0.83, 1.17) | 0.86 (0.72, 1.02) | 0.87 (0.72, 1.05) |
| Model 3^c^, HR (95% CI) | 1 (reference) | 0.95 (0.80, 1.12) | 0.83 (0.69, 0.99) | 0.84 (0.69, 1.01) |
| **Animal-based sources** |  |  |  |  |
| Mean intake (SD), mg/d | 15.70 (15.65) | 72.94 (16.32) | 137.40 (22.23) | 308.67 (267.52) |
| Events/participants | 352/2578 | 296/2579 | 261/2575 | 241/2578 |
| Model 1^a^, HR (95% CI) | 1 (reference) | 0.99 (0.85, 1.15) | 0.98 (0.83, 1.15) | 0.91 (0.77, 1.07) |
| Model 2^b^, HR (95% CI) | 1 (reference) | 0.91 (0.77, 1.07) | 0.95 (0.79, 1.14) | 0.81 (0.68, 1.00) |
| Model 3^c^, HR (95% CI) | 1 (reference) | 0.93 (0.79, 1.10) | 0.95 (0.80, 1.14) | 0.85 (0.69, 1.06) |
| **Pre-frailty (n=6969)** |  |  |  |  |
| **Plant-based sources** |  |  |  |  |
| Mean intake (SD), mg/d | 40.07 (10.57) | 69.41 (8.24) | 104.66 (12.90) | 206.79 (110.16) |
| Events/participants | 573/1743 | 561/1742 | 600/1742 | 642/1742 |
| Model 1^a^, HR (95% CI) | 1 (reference) | 0.92 (0.82, 1.04) | 0.94 (0.84, 1.05) | 0.95 (0.85, 1.06) |
| Model 2^b^, HR (95% CI) | 1 (reference) | 0.90 (0.80, 1.02) | 0.93 (0.82, 1.05) | 0.97 (0.84, 1.10) |
| Model 3^c^, HR (95% CI) | 1 (reference) | 0.90 (0.80, 1.01) | 0.91 (0.80, 1.03) | 0.95 (0.83, 1.08) |
| **Animal-based sources** |  |  |  |  |
| Mean intake (SD), mg/d | 16.15 (16.08) | 74.05 (16.28) | 137.04 (22.07) | 304.63 (196.52) |
| Events/participants | 717/1750 | 584/1735 | 527/1742 | 548/1742 |
| Model 1^a^, HR (95% CI) | 1 (reference) | 0.91 (0.81, 1.01) | 0.89 (0.79, 1.00) | 0.94 (0.84, 1.05) |
| Model 2^b^, HR (95% CI) | 1 (reference) | 0.91 (0.81, 1.03) | 0.91 (0.80, 1.03) | 0.95 (0.82, 1.09) |
| Model 3^c^, HR (95% CI) | 1 (reference) | 0.92 (0.82, 1.03) | 0.90 (0.79, 1.02) | 0.95 (0.83, 1.10) |
| **(Pre-) frailty* (n=7222)** |  |  |  |  |
| **Plant-based sources** |  |  |  |  |
| Mean intake (SD), mg/d | 39.93 (10.54) | 69.21 (8.16) | 104.23 (12.86) | 205.92 (109.07) |
| Events/participants | 644/1806 | 631/1805 | 658/1805 | 696/1806 |
| Model 1^a^, HR (95% CI) | 1 (reference) | 0.92 (0.83, 1.03) | 0.92 (0.82, 1.02) | 0.92 (0.82, 1.02) |
| Model 2^b^, HR (95% CI) | 1 (reference) | 0.92 (0.82, 1.04) | 0.93 (0.82, 1.05) | 0.95 (0.84, 1.08) |
| Model 3^c^, HR (95% CI) | 1 (reference) | 0.92 (0.82, 1.03) | 0.92 (0.81, 1.03) | 0.94 (0.82, 1.06) |
| **Animal-based sources** |  |  |  |  |
| Mean intake (SD), mg/d | 15.79 (15.79) | 73.44 (16.51) | 136.76 (21.92) | 303.89 (194.81) |
| Events/participants | 790/1806 | 639/1805 | 595/1805 | 605/1806 |
| Model 1^a^, HR (95% CI) | 1 (reference) | 0.89 (0.80, 0.98) | 0.90 (0.81, 1.00) | 0.93 (0.84, 1.04) |
| Model 2^b^, HR (95% CI) | 1 (reference) | 0.90 (0.80, 1.00) | 0.92 (0.82, 1.03) | 0.94 (0.82, 1.08) |
| Model 3^c^, HR (95% CI) | 1 (reference) | 0.91 (0.81, 1.01) | 0.91 (0.81, 1.03) | 0.95 (0.83, 1.09) |

* The term (pre-) frailty refers to the combined states of pre-frailty and frailty.

^a^ The crude model was not adjusted for any confounders.

^b^ Adjusted for age (years, continuous), sex (female or male), nationality (Han or minority), residence (urban or rural), geographical region (northeastern China, eastern China, central China, southern China or southwestern China), marital status (married, single, or divorced, widowed or separated), education (at or below primary school, middle school, or at or above high school), household per capita annual income (CNY, continuous), medical insurance (yes or no), drinking (never, moderate, or excess), smoking (never, previous, or current), drinking water source (tap water, well water or other), cooking fuel type (clean fuel or polluting fuel), sedentary behavior (hours/d, continuous), dietary intake of energy (kcal/d, continuous), protein (g/d, continuous), fat (g/d, continuous), and carbohydrate (g/d, continuous).

^c^ Additionally adjusted for body mass index (kg/m^2^, continuous), hypertension (yes or no), health infrastructure score (continuous), sanitation score (continuous), and social services score (continuous).

| **Table S7. Associations of total choline intake with incident frailty: subgroup analysis stratified by sex, age and energy intake** | | | | | |
| --- | --- | --- | --- | --- | --- |
| **Subgroup** | **Total choline intake quartiles** | | | | ***P* for interaction** |
|  | **1** | **2** | **3** | **4** |  |
| **Sex** |  |  |  |  | 0.3318 |
| Male | 1 (reference) | 0.76 (0.59, 0.98) | 0.75 (0.57, 0.99) | 0.68 (0.50, 0.95) |  |
| Female | 1 (reference) | 0.88 (0.71, 1.09) | 0.77 (0.61, 0.98) | 0.82 (0.62, 1.07) |  |
| **Age** |  |  |  |  | 0.1962 |
| 18-44 | 1 (reference) | 0.80 (0.49, 1.31) | 1.08 (0.67, 1.75) | 1.05 (0.61, 1.81) |  |
| 45-59 | 1 (reference) | 0.90 (0.70, 1.15) | 0.87 (0.66, 1.15) | 0.74 (0.54, 1.03) |  |
| ≥ 60 | 1 (reference) | 0.80 (0.62, 1.02) | 0.72 (0.54, 0.94) | 0.75 (0.55, 1.04) |  |
| **Energy intake** |  |  |  |  | 0.9242 |
| T1 | 1 (reference) | 0.93 (0.70, 1.22) | 0.81 (0.60, 1.10) | 0.71 (0.50, 1.02) |  |
| T2 | 1 (reference) | 0.76 (0.56, 1.02) | 0.91 (0.66, 1.25) | 0.81 (0.55, 1.17) |  |
| T3 | 1 (reference) | 0.94 (0.71, 1.26) | 0.81 (0.60, 1.11) | 0.90 (0.63, 1.29) |  |

Adjusted for age (years, continuous), sex (female or male), nationality (Han or minority), residence (urban or rural), geographical region (northeastern China, eastern China, central China, southern China or southwestern China), marital status (married, single, or divorced, widowed or separated), education (at or below primary school, middle school, or at or above high school), household per capita annual income (CNY, continuous), medical insurance (yes or no), drinking (never, moderate, or excess), smoking (never, previous, or current), drinking water source (tap water, well water or other), cooking fuel type (clean fuel or polluting fuel), sedentary behavior (hours/d, continuous), dietary intake of energy (kcal/d, continuous), protein (g/d, continuous), fat (g/d, continuous), and carbohydrate (g/d, continuous), body mass index (kg/m^2^, continuous), hypertension (yes or no), health infrastructure score (continuous), sanitation score (continuous), and social services score (continuous).

| **Table S8. Associations of total choline with incident** **pre-frailty: subgroup analysis stratified by sex, age and energy intake** | | | | | |
| --- | --- | --- | --- | --- | --- |
| **Subgroup** | **Total choline intake quartiles** | | | | ***P* for interaction** |
|  | **1** | **2** | **3** | **4** |  |
| **Sex** |  |  |  |  | 0.0546 |
| Male | 1 (reference) | 0.84 (0.71, 1.00) | 0.87 (0.72, 1.05) | 0.94 (0.76, 1.16) |  |
| Female | 1 (reference) | 0.99 (0.85, 1.16) | 0.88 (0.74, 1.04) | 0.96 (0.80, 1.17) |  |
| **Age** |  |  |  |  | 0.0568 |
| 18-44 | 1 (reference) | 0.93 (0.76, 1.14) | 0.94 (0.76, 1.16) | 1.07 (0.84, 1.35) |  |
| 45-59 | 1 (reference) | 0.91 (0.75, 1.11) | 0.85 (0.69, 1.04) | 0.96 (0.76, 1.22) |  |
| ≥ 60 | 1 (reference) | 1.20 (0.92, 1.56) | 0.98 (0.74, 1.31) | 1.28 (0.93, 1.75) |  |
| **Energy intake** |  |  |  |  | 0.0758 |
| T1 | 1 (reference) | 0.92 (0.75, 1.13) | 0.83 (0.67, 1.04) | 0.85 (0.66, 1.11) |  |
| T2 | 1 (reference) | 1.07 (0.88, 1.31) | 1.00 (0.81, 1.25) | 1.17 (0.92, 1.49) |  |
| T3 | 1 (reference) | 0.86 (0.70, 1.05) | 0.82 (0.67, 1.02) | 0.96 (0.76, 1.21) |  |

Adjusted for age (years, continuous), sex (female or male), nationality (Han or minority), residence (urban or rural), geographical region (northeastern China, eastern China, central China, southern China or southwestern China), marital status (married, single, or divorced, widowed or separated), education (at or below primary school, middle school, or at or above high school), household per capita annual income (CNY, continuous), medical insurance (yes or no), drinking (never, moderate, or excess), smoking (never, previous, or current), drinking water source (tap water, well water or other), cooking fuel type (clean fuel or polluting fuel), sedentary behavior (hours/d, continuous), dietary intake of energy (kcal/d, continuous), protein (g/d, continuous), fat (g/d, continuous), and carbohydrate (g/d, continuous), body mass index (kg/m^2^, continuous), hypertension (yes or no), health infrastructure score (continuous), sanitation score (continuous), and social services score (continuous).

| **Table S9. Associations of total choline intake with incident (pre-) frailty*: subgroup analysis stratified by sex, age and energy intake** | | | | | |
| --- | --- | --- | --- | --- | --- |
| **Subgroup** | **Total choline intake quartile** | | | | ***P* for interaction** |
|  | **1** | **2** | **3** | **4** |  |
| **Sex** |  |  |  |  | 0.0899 |
| Male | 1 (reference) | 0.82 (0.70, 0.97) | 0.87 (0.73, 1.04) | 0.92 (0.76, 1.13) |  |
| Female | 1 (reference) | 1.00 (0.86, 1.16) | 0.88 (0.75, 1.03) | 0.96 (0.80, 1.16) |  |
| **Age** |  |  |  |  | 0.0731 |
| 18-44 | 1 (reference) | 0.90 (0.74, 1.09) | 0.91 (0.74, 1.11) | 1.04 (0.83, 1.31) |  |
| 45-59 | 1 (reference) | 0.99 (0.84, 1.16) | 0.81 (0.68, 0.97) | 0.82 (0.67, 1.01) |  |
| ≥ 60 | 1 (reference) | 1.22 (0.96, 1.55) | 0.98 (0.76, 1.27) | 1.27 (0.95, 1.70) |  |
| **Energy intake** |  |  |  |  | 0.0377 |
| T1 | 1 (reference) | 0.98 (0.81, 1.19) | 0.82 (0.67, 1.02) | 0.87 (0.68, 1.11) |  |
| T2 | 1 (reference) | 1.03 (0.85, 1.25) | 1.00 (0.81, 1.24) | 1.15 (0.91, 1.45) |  |
| T3 | 1 (reference) | 0.87 (0.72, 1.05) | 0.84 (0.69, 1.03) | 0.98 (0.79, 1.22) |  |

* The term (pre-) frailty refers to the combined states of pre-frailty and frailty.

Adjusted for age (years, continuous), sex (female or male), nationality (Han or minority), residence (urban or rural), geographical region (northeastern China, eastern China, central China, southern China or southwestern China), marital status (married, single, or divorced, widowed or separated), education (at or below primary school, middle school, or at or above high school), household per capita annual income (CNY, continuous), medical insurance (yes or no), drinking (never, moderate, or excess), smoking (never, previous, or current), drinking water source (tap water, well water or other), cooking fuel type (clean fuel or polluting fuel), sedentary behavior (hours/d, continuous), dietary intake of energy (kcal/d, continuous), protein (g/d, continuous), fat (g/d, continuous), and carbohydrate (g/d, continuous), body mass index (kg/m^2^, continuous, hypertension (yes or no), health infrastructure score (continuous), sanitation score (continuous), and social services score (continuous).

| **Table S10. Associations of total choline intake with incident frailty: sensitivity analysis** | | | | |
| --- | --- | --- | --- | --- |
| **Sensitivity analysis** | **Total choline intake quartiles** | | | |
|  | **1** | **2** | **3** | **4** |
| **Exclude participants who experienced frailty in the initial 2 years of follow-up (n=10065)** |  |  |  |  |
| Events/participants | 293/2517 | 211/2516 | 216/2516 | 185/2516 |
| Model 1^a^, HR (95% CI) | 1 (reference) | 0.79 (0.67, 0.95) | 0.86 (0.72, 1.02) | 0.75 (0.63, 0.91) |
| Model 2^b^, HR (95% CI) | 1 (reference) | 0.82 (0.68, 0.98) | 0.82 (0.68, 1.00) | 0.73 (0.57, 0.92) |
| Model 3^c^, HR (95% CI) | 1 (reference) | 0.81 (0.67, 0.97) | 0.83 (0.68, 1.02) | 0.73 (0.58, 0.93) |
| **Exclude participants who experienced frailty in the initial 4 years of follow-up (n=9864)** |  |  |  |  |
| Events/participants | 231/2466 | 165/2466 | 174/2466 | 134/2466 |
| Model 1^a^, HR (95% CI) | 1 (reference) | 0.81 (0.66, 0.99) | 0.91 (0.75, 1.11) | 0.72 (0.58, 0.89) |
| Model 2^b^, HR (95% CI) | 1 (reference) | 0.79 (0.64, 0.97) | 0.81 (0.65, 1.00) | 0.59 (0.45, 0.77) |
| Model 3^c^, HR (95% CI) | 1 (reference) | 0.77 (0.63, 0.95) | 0.81 (0.65, 1.01) | 0.59 (0.45, 0.78) |
| **Exclude participants who with (pre-) frailty* baseline (n=7222)** |  |  |  |  |
| Events/participants | 149/1806 | 105/1805 | 98/1805 | 87/1806 |
| Model 1^a^, HR (95% CI) | 1 (reference) | 0.78 (0.61, 1.00) | 0.76 (0.59, 0.98) | 0.70 (0.54, 0.91) |
| Model 2^b^, HR (95% CI) | 1 (reference) | 0.81 (0.62, 1.05) | 0.74 (0.56, 0.98) | 0.67 (0.48, 0.93) |
| Model 3^c^, HR (95% CI) | 1 (reference) | 0.80 (0.62, 1.04) | 0.76 (0.57, 1.00) | 0.68 (0.49, 0.96) |
| **Additionally adjusted for survey year in each model (n=10310)** |  |  |  |  |
| Events/participants | 368/2578 | 276/2577 | 264/2577 | 242/2578 |
| Model 1^a^, HR (95% CI) | 1 (reference) | 0.80 (0.69, 0.94) | 0.79 (0.68, 0.93) | 0.74 (0.63, 0.87) |
| Model 2^b^, HR (95% CI) | 1 (reference) | 0.84 (0.72, 0.99) | 0.79 (0.66, 0.94) | 0.75 (0.61, 0.93) |
| Model 3^c^, HR (95% CI) | 1 (reference) | 0.83 (0.71, 0.98) | 0.80 (0.67, 0.95) | 0.75 (0.61, 0.93) |
| **Residual energy-adjusted total choline intake as exposure (n=10310)** |  |  |  |  |
| Events/participants | 355/2578 | 276/2577 | 281/2577 | 238/2578 |
| Model 1^a^, HR (95% CI) | 1 (reference) | 0.88 (0.75, 1.03) | 0.99 (0.84, 1.15) | 0.86 (0.73, 1.02) |
| Model 2^b^, HR (95% CI) | 1 (reference) | 0.77 (0.65, 0.91) | 0.83 (0.70, 1.00) | 0.76 (0.62, 0.93) |
| Model 3^c^, HR (95% CI) | 1 (reference) | 0.76 (0.64, 0.90) | 0.84 (0.70, 1.00) | 0.78 (0.64, 0.96) |
| **Additionally adjusted for lipid profiles: MICE-imputed dataset (n=10310)** |  |  |  |  |
| Events/participants | 368/2578 | 276/2577 | 264/2577 | 242/2578 |
| Model 1^d^, HR (95% CI) | 1 (reference) | 0.84 (0.71, 0.99) | 0.80 (0.67, 0.96) | 0.76 (0.62, 0.94) |
| Model 2^e^, HR (95% CI) | 1 (reference) | 0.86 (0.73, 1.02) | 0.80 (0.67, 0.95) | 0.79 (0.64, 0.97) |
| **Additionally adjusted for lipid profiles: original dataset (n=5876)** |  |  |  |  |
| Events/participants | 269/1469 | 191/1469 | 217/1469 | 171/1469 |
| Model 1^d^, HR (95% CI) | 1 (reference) | 0.74 (0.61, 0.90) | 0.86 (0.70, 1.04) | 0.71 (0.56, 0.91) |
| Model 2^e^, HR (95% CI) | 1 (reference) | 0.78 (0.64, 0.95) | 0.87 (0.71, 1.06) | 0.75 (0.58, 0.95) |

* The term (pre-) frailty refers to the combined states of pre-frailty and frailty.

^a^ The crude model was not adjusted for any confounders.

^b^ Adjusted for age (years, continuous), sex (female or male), nationality (Han or minority), residence (urban or rural), geographical region (northeastern China, eastern China, central China, southern China or southwestern China), marital status (married, single, or divorced, widowed or separated), education (at or below primary school, middle school, or at or above high school), household per capita annual income (CNY, continuous), medical insurance (yes or no), drinking (never, moderate, or excess), smoking (never, previous, or current), drinking water source (tap water, well water or other), cooking fuel type (clean fuel or polluting fuel), sedentary behavior (hours/d, continuous), dietary intake of energy (kcal/d, continuous), protein (g/d, continuous), fat (g/d, continuous), and carbohydrate (g/d, continuous).

^c^ Additionally adjusted for body mass index (kg/m^2^, continuous), hypertension (yes or no), health infrastructure score (continuous), sanitation score (continuous), and social services score (continuous).

^d^ Additionally adjusted for hyperlipidemia (yes or no).

^e^ Adjusted for age (years, continuous), sex (female or male), nationality (Han or minority), residence (urban or rural), geographical region (northeastern China, eastern China, central China, southern China or southwestern China), marital status (married, single, or divorced, widowed or separated), education (at or below primary school, middle school, or at or above high school), household per capita annual income (CNY, continuous), medical insurance (yes or no), drinking (never, moderate, or excess), smoking (never, previous, or current), drinking water source (tap water, well water or other), cooking fuel type (clean fuel or polluting fuel), sedentary behavior (hours/d, continuous), dietary intake of energy (kcal/d, continuous), protein (g/d, continuous), fat (g/d, continuous), and carbohydrate (g/d, continuous), body mass index (kg/m^2^, continuous), health infrastructure score (continuous), sanitation score (continuous), social services score (continuous), systolic blood pressure (mmHg, continuous), diastolic blood pressure (mmHg, continuous), total cholesterol (mmol/L, continuous), triglycerides (mmol/L, continuous), and high-density lipoprotein cholesterol (mmol/L, continuous).

**Table S11. Characteristics of participants for lipid profiles**

| **Lipid profiles** | **Total** | **Total choline intake quartiles** | | | |
| --- | --- | --- | --- | --- | --- |
|  |  | **1** | **2** | **3** | **4** |
| **MICE-imputed dataset** |  |  |  |  |  |
| Participants, No. (%) | 10310 (100.00) | 2578 (25.00) | 2577 (25.00) | 2577 (25.00) | 2578 (25.00) |
| Hyperlipidemia, No. (%) | 3657 (35.47) | 864 (33.51) | 892 (34.61) | 944 (36.63) | 957 (37.12) |
| TC (mmol/L), median (25^th^, 75^th^) | 4.78 (4.18, 5.46) | 4.80 (4.24, 5.46) | 4.77 (4.19, 5.44) | 4.78 (4.16, 5.46) | 4.76 (4.14, 5.48) |
| TG (mmol/L), median (25^th^, 75^th^) | 1.28 (0.86, 2.01) | 1.28 (0.86, 1.97) | 1.26 (0.85, 1.92) | 1.29 (0.88, 2.01) | 1.30 (0.87, 2.14) |
| HDL-C (mmol/L), median (25^th^, 75^th^) | 1.39 (1.16, 1.66) | 1.41 (1.18, 1.68) | 1.40 (1.18, 1.66) | 1.37 (1.15, 1.65) | 1.36 (1.15, 1.62) |
| LDL-C (mmol/L), median (25^th^, 75^th^) | 2.91 (2.34, 3.53) | 2.91 (2.33, 3.54) | 2.91 (2.35, 3.54) | 2.92 (2.36, 3.52) | 2.87 (2.31, 3.54) |
| **Original dataset** |  |  |  |  |  |
| Participants, No. (%) | 5876 (100.00) | 1469 (25.00) | 1469 (25.00) | 1469 (25.00) | 1469 (25.00) |
| Hyperlipidemia, No. (%) | 1956 (33.29) | 461 (31.38) | 453 (30.84) | 512 (34.83) | 530 (36.08) |
| TC (mmol/L), median (25^th^, 75^th^) | 4.77 (4.17, 5.45) | 4.79 (4.20, 5.45) | 4.76 (4.16, 5.40) | 4.78 (4.15, 5.45) | 4.78 (4.18, 5.49) |
| TG (mmol/L), median (25^th^, 75^th^) | 1.25 (0.85, 1.92) | 1.24 (0.83, 1.84) | 1.22 (0.83, 1.85) | 1.25 (0.87, 1.91) | 1.28 (0.85, 2.06) |
| HDL-C (mmol/L), median (25^th^, 75^th^) | 1.39 (1.17, 1.64) | 1.42 (1.19, 1.67) | 1.40 (1.18, 1.66) | 1.38 (1.15, 1.63) | 1.36 (1.14, 1.62) |
| LDL-C (mmol/L), median (25^th^, 75^th^) | 2.91 (2.34, 3.53) | 2.92 (2.33, 3.51) | 2.86 (2.33, 3.49) | 2.93 (2.36, 3.53) | 2.90 (2.35, 3.55) |

TC: Total cholesterol, TG: Triglyceride, HDL-C: High-density lipoprotein cholesterol, and LDL-C: Low-density lipoprotein cholesterol.

**Supplement Reference**

S1. Zhang B, Zhai FY, Du SF, Popkin BM. The China Health and Nutrition Survey, 1989-2011. Obes Rev 2014; 15 Suppl 1(0 1): 2-7.

S2. Pan F, Wang Z, Wang H, et al. Association between Free Sugars Intake and Risk of Metabolic Syndrome in Chinese Adults: Results from the China Health and Nutrition Survey, 2000-2018. Nutrients 2022; 14(24).

S3. Kiss N, Abbott G, Daly RM, et al. Multimorbidity and the risk of malnutrition, frailty and sarcopenia in adults with cancer in the UK Biobank. J Cachexia Sarcopenia Muscle 2024; 15(5): 1696-707.

S4. Qiu W, Cai A, Li L, Feng Y. Longitudinal Trajectories of Alcohol Consumption with All-Cause Mortality, Hypertension, and Blood Pressure Change: Results from CHNS Cohort, 1993-2015. Nutrients 2022; 14(23).

S5. Organization W.H. World Health Organization; 2000. International guide for monitoring alcohol consumption and related harm.

S6. Li X, Duan C, Chen Q, Xiao J, Jim Zhang J. Associations between cooking fuels and hypertension prevalence in Chinese adults: A prospective cohort analysis focusing on fuel transitioning. Environ Int 2023; 175: 107953.

S7. Institute for Nutrition and Food Safety of the Chinese Center for Disease Control and Prevention China Food Composition Table 2004. 1st ed Beijing, China, Peking University Medical Press, 2005.

S8. Institute for Nutrition and Food Safety of the Chinese Center for Disease Control and Prevention China Food Composition Table. Beijing, China, Peking University Medical Press, 2002.

S9. Jones-Smith JC, Popkin BM. Understanding community context and adult health changes in China: development of an urbanicity scale. Soc Sci Med 2010; 71(8): 1436-46.

S10. Xia PF, Zhang YB, Liu G, Pan A. [The application of energy adjustment models in nutritional epidemiology]. Zhonghua Yu Fang Yi Xue Za Zhi 2020; 54(2): 228-32.

S11. Fischer LM, daCosta KA, Kwock L, et al. Sex and menopausal status influence human dietary requirements for the nutrient choline. Am J Clin Nutr 2007; 85(5): 1275-85.

S12. Fang Y, Vilella-Bach M, Bachmann R, Flanigan A, Chen J. Phosphatidic acid-mediated mitogenic activation of mTOR signaling. Science 2001; 294(5548): 1942-5.

S13. Ng SW, Norton EC, Popkin BM. Why have physical activity levels declined among Chinese adults? Findings from the 1991-2006 China Health and Nutrition Surveys. Soc Sci Med 2009; 68(7): 1305-14.

S14. Zuo H, Shi Z, Yuan B, et al. Interaction between physical activity and sleep duration in relation to insulin resistance among non-diabetic Chinese adults. BMC Public Health 2012; 12: 247.

S15. Su Y, Li X, Li H, Xu J, Xiang M. Association between Sedentary Behavior during Leisure Time and Excessive Weight in Chinese Children, Adolescents, and Adults. Nutrients 2023; 15(2).

S16. Yan M, Liu Y, Wu L, et al. The Association between Dietary Purine Intake and Mortality: Evidence from the CHNS Cohort Study. Nutrients 2022; 14(9).

S17. Jones-Smith JC, Popkin BM. Understanding community context and adult health changes in China: development of an urbanicity scale. Soc Sci Med 2010; 71(8): 1436-46.

S18. Zou Q, Su C, Du W, et al. The Mediation and Moderation Effect Association among Physical Activity, Body-Fat Percentage, Blood Pressure, and Serum Lipids among Chinese Adults: Findings from the China Health and Nutrition Surveys in 2015. Nutrients 2023; 15(14).

S19. Wang J, Shi T, Xu L, et al. Correlation between hyperlipidemia and serum vitamin D levels in an adult Chinese cohort. Front Nutr 2024; 11: 1302260
